# Reassessing CORM-A1: redox chemistry and idiosyncratic CO-releasing characteristics of the widely used carbon monoxide donor†

**DOI:** 10.1039/d3sc00411b

**Published:** 2023-02-24

**Authors:** Nicola Bauer, Xiaoxiao Yang, Zhengnan Yuan, Binghe Wang

**Affiliations:** a Department of Chemistry and Center for Diagnostics and Therapeutics, Georgia State University Atlanta Georgia 30303 USA wang@gsu.edu

## Abstract

Carbon monoxide (CO) is an endogenous signaling molecule with demonstrated ability to modulate immune responses and to engage key components of the circadian clock. Further, CO has been pharmacologically validated for its therapeutic benefits in animal models of various pathological conditions. For the development of CO-based therapeutics, new delivery forms are needed to address the inherent limitations of using inhaled CO for therapeutic applications. Along this line, there have been metal– and borane–carbonyl complexes reported as CO-release molecules (CORMs) for various studies. CORM-A1 is among the four most widely used CORMs in examining CO biology. Such studies are predicated on the assumptions that CORM-A1 (1) releases CO efficiently and reproducibly under commonly used experimental conditions and (2) does not have meaningful CO-independent activities. In this study, we demonstrate the important redox properties of CORM-A1 leading to the reduction of bio-relevant molecules such as NAD^+^ and NADP^+^ under near-physiological conditions; such reduction reciprocally facilitates CO release from CORM-A1. We further demonstrate that CO-release yield and rate from CORM-A1 are highly dependent on various factors such as the medium used, buffer concentrations, and redox environment; these factors seem to be so idiosyncratic that we were unable to find a uniform mechanistic explanation. Under standard experimental conditions, CO release yields were found to be low and highly variable (0.5–15%) in the initial 15 min unless in the presence of certain reagents, *e.g.* NAD^+^ or high concentrations of buffer. The significant chemical reactivity of CORM-A1 and the highly variable nature of CO release under near-physiological conditions suggest the need for much more consideration of appropriate controls, if available, and caution in using CORM-A1 as a CO surrogate in biological studies.

## Abstract 2

Redox activity and unreliable carbon monoxide production of CO donor, CORM-A1, presents new complications in its use for studying CO biology.

## Graphical abstract

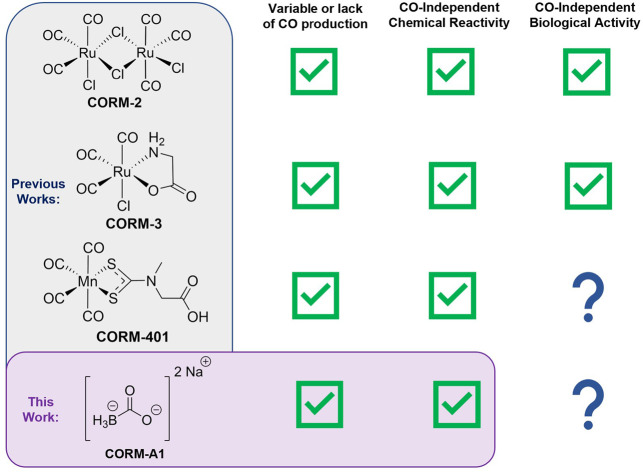

## Introduction

**Fig. 1:**
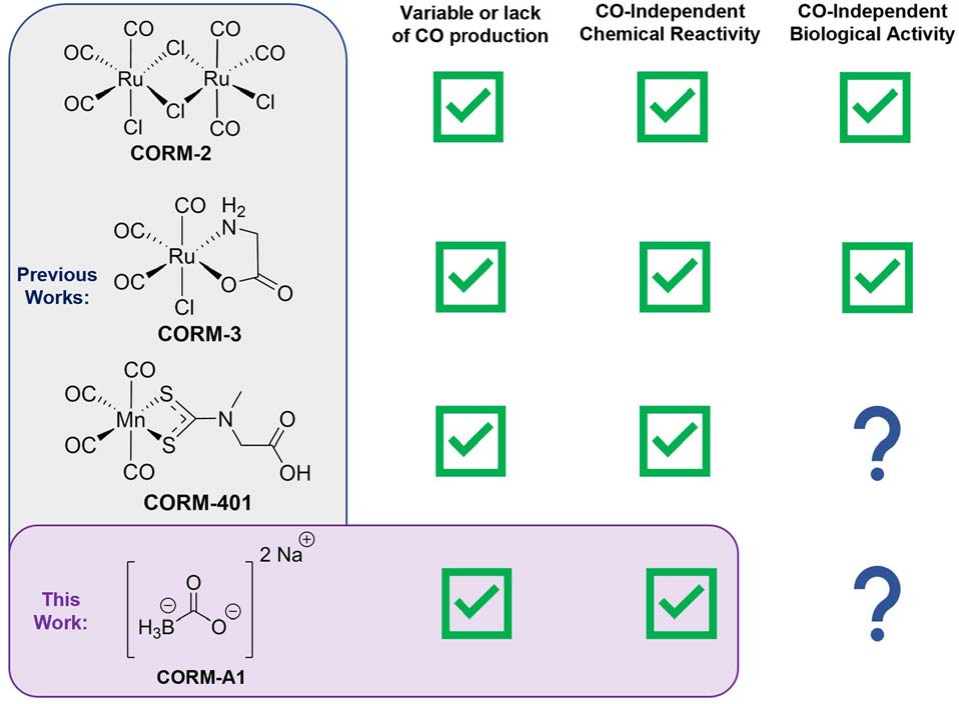
(Blue) Structures of CORM-2, CORM-3, and CORM-401 and brief overview of their reported CO-independent biological and chemical reactivities and (Purple) structure of CORM-A1 and an overview of the work presented here.

Carbon monoxide (CO) is an endogenous signaling molecule with functional importance on par with that of two other gaseous signaling molecules: nitric oxide and hydrogen sulfide.^1–3^ CO is produced in mammals primarily through heme degradation mediated by heme-oxygenase (HO). Exogenous CO has been pharmacologically validated to provide therapeutic benefits in animal models of ischemia-reperfusion injury of various organs, organ transplantations, anti-inflammation, and even cancer. A recent book comprehensively summarizes CO biology and its therapeutic potential including clinical trials.^4^ Early studies of CO biology mostly used gaseous CO, which has the advantage of little ambiguity in terms of the active principal and the disadvantage of difficulty in controlling dosage and concentration, lack of portability, health hazards to lab personnel, and dosage variations depending on individual characteristics such as lung capacity, breathing rate, and physiological state.^5^ The last point is especially problematic if CO-based therapeutics ever go into human studies. To address these issues, new forms of CO have been developed including metal-based CO-releasing molecules (CORMs),^6–9^ and organic CO donors capable of releasing CO upon photolysis, mechanical force, or chemoexcitation.^10–15^ There have also been efforts to use various triggers for CO release from metal-based CORMs.^16–20^ In 2014, we reported the first organic CO prodrugs by taking advantage of a cheletropic reaction for CO release from a norbornadienone scaffold; this was followed by a series of reports of CO prodrugs of various properties^21–24^ including one that uses saccharine and acesulfame as carrier molecules for CO delivery.^25^ Among the large number of CO donors published, CORM-2, CORM-3, CORM-401, and CORM-A1 are probably the most well-known (Fig. 1).^6,26,27^ Due to their commercial availability and ease of use, these four CORMs have been widely used as CO surrogates in a large number of studies examining the biological effects of CO.^27–29^ Combined, they had appeared in about 650 publications, based on a Pubmed search in January 2023. Recently, extensive CO-independent chemical reactivities for these molecules have been reported that could impact the interpretation of results from using these CORMs. Specifically, ruthenium-based molecules CORM-2 and CORM-3 have been shown to react with nucleophiles such as the thiol group on cysteine and the imidazole group in histidine and to have catalase-like, redox, and radical scavenging activity.^30–34^ Under near-physiological conditions, aqueous solutions of CORM-2 and CORM-3 have been shown to mostly produce CO_2_ instead of CO, through an redox reaction.^30,31,35^ Along a similar line, CORM-401 has been shown to react with reactive oxygen species (ROS) such as hydrogen peroxide and free radicals, which are commonly recognized as key mediators in CO's signaling actions.^30^ Similarly, CO-independent biological activities have been reported by various studies.^32–34,36–38^ Incidentally, some of these CORMs (CORM-2, CORM-3) have been used as the sole CO source in developing CO-sensing methods, leading to “CO probes” that fail to detect CO and only detect the CORM used.

**Scheme 1:**
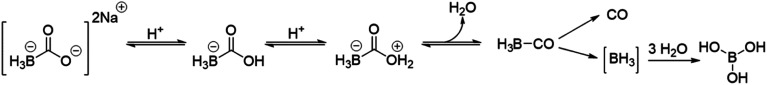
Proposed CO release mechanism from CORM-A1 as reported by Motterlini and coworkers.^26^

Based on an earlier literature report of a borane based compound capable of CO release in aqueous solutions, Motterlini and coworkers proposed its use as a CO surrogate for studying CO biology and pharmacology and named the compound CORM-A1.^26^ The mechanism of CO release was described as decomposition upon protonation to liberate CO spontaneously *via* an unstable borane carbonyl intermediate (Scheme 1).^26^ This mechanism is distinctly different from other metal-based CORMs such as CORM-2 and CORM-3, which dissociate CO through light irradiation or ligand substitution.^6,30,31^ Further, if BH_3_ is an intermediate, it is expected to either release molecular hydrogen upon reaction with reagents with active protons (*e.g.* H_2_O) or undergo oxidation reactions with an “oxidizing partner,” though this was not part of the originally proposed mechanistic aspect. Due to its commercially availability, solubility in water, lack of a transition metal, and stated fast CO release rate (*t*_1/2_, 2–21 min), CORM-A1 has been widely used in biological studies as a CO donor with the assumption that it is benign otherwise and donates near stoichiometric amounts of CO with the release half-life as indicated in the original report.^26,28,39,40^

In our own work of developing CO prodrugs for various applications, we were frequently asked to compare against a widely used CORM as a “positive control.”^22^ Therefore, we took an interest in examining CORM-2, CORM-3, CORM-401, and CORM-A1, which were considered as the “standard” positive controls. We have already conducted extensive studies of the first three (Fig. 1). Specific to this study, we examine CORM-A1. A search of the term “CORM-A1” in Pubmed in January 2023 yielded 79 publications covering studies examining CO's effects on autophagy, coagulation/platelet activation, non-alcoholic steatohepatitis, myocardial infarction, neuronal differentiation, blood–brain barrier dysfunction, cancer metastasis, autoimmune uveoretinitis, inflammation and fibrosis, and neonatal vascular injury, among many other indications. Such a broad range of activities and the large number of publications using CORM-A1 clearly indicate its broad impact in the CO field. However, among these four widely used CORMs, CORM-A1 is the least characterized in terms of CO release properties and chemical reactivity, despite the presence of a BH_3_ moiety, which is known to be chemically reactive. Further, reports of CO-independent biological activity are emerging. Therefore, we took an interest in examining the chemical reactivity of CORM-A1 and its CO production characteristics under various conditions. To us, a viable CO surrogate for studying CO biology and pharmacology should meet three basic criteria. First, the “surrogate” should not have pronounced chemical reactivity toward biomolecules commonly seen in cellular functions or *in vivo.* Second, there is a good negative control of the “carrier” portion of the delivery system; in the case of a CORM, the CO-depleted product (referred to as iCORM) is commonly considered as the most acceptable negative control. Lastly, and probably most importantly: it should be able to reproducibly generate near stoichiometric amount of CO under near physiological conditions with well-defined kinetics and well-established release profiles. Further, factors that could affect the CO release yield and kinetics should be well understood within the confines of commonly-encountered variations in lab experiments and *in vivo.* Herein, we describe our work in examining CORM-A1 along these three lines, starting with the examination of chemical reactivities because these properties directly impact both CO yields and the analysis of the issue of a negative control (Fig. 1).

## Results and discussion

### Criterion I. The chemical reactivity of CORM-A1 towards biorelevant molecules

**Fig. 2:**
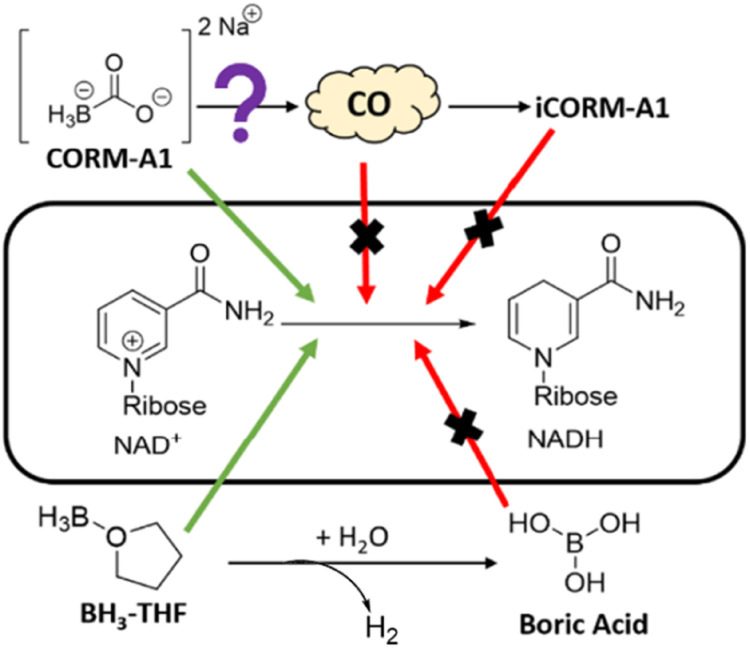
Investigation of NAD^+^ reduction by CORM-A1.

CORM-A1 is a complex of borane, which is a textbook case of a common reducing agent for a wide range of functional groups and molecules. Though, the presence of a borane moiety in CORM-A1 is strongly indicative of its reducing power and suggests the need to carefully examine this issue, it is not readily predictable, at least not conclusively, as to whether this specific complex would be reactive enough to pose a reactivity issue in solution and in biological milieu. Earlier, we have reported the ability for CORM-A1 to react and consume H_2_O_2_ and free radicals, which are very important in redox signalling of CO.^30^ Therefore, initial indications are that the direct reactivity of CORM-A1 could pose a challenge to the examination of CO's roles in various redox signalling processes when it is used as a CO surrogate. In this study, we are interested in examining the direct reaction of CORM-A1 with biomolecules important to cellular functions. Among the different possibilities, we chose to study the effect of CORM-A1 on NAD^+^ and NADP^+^, because of their widely recognized roles in essential cellular processes (Fig. 2). Further, changes in the concentration of NAD^+^ and the ratio of NAD^+^/NADH have been specifically implicated in CO's roles in regulating platelet activation and energy metabolism.^28,29^ Again, CORM-A1 has structural similarity with other BH_3_ complexes and borohydride reagents such as sodium borohydride and sodium cyanoborohydride, which are known to reduce *N*-methylated nicotinamides.^41,42^ Therefore, it is important to study the reactivity issue of CORM-A1 in redox processes either to eliminate such reactivity as a confounding factor in result interpretation or to raise caution in using CORM-A1 as a pure “CO surrogate.” In doing so, we focused on three key aspects as shown in Fig. 2. First, is there significant chemical reactivity between CORM-A1 and NAD^+^ or NADP^+^ respectively? If so, is it the borane moiety that is responsible for the observed reduction? Secondly, does the CO-depleted product (commonly referred to as iCORM) serve as an adequate control based on chemical reactivity? Boric acid is sometimes used as a negative control for CORM-A1. Is it an adequate control based on chemical reactivity? Thirdly, does the reaction between CORM-A1 and NAD^+^ or NADP^+^ have any impact on the CO-releasing property of CORM-A1? Below we describe our findings and analyses.

#### CORM-A1 reduces NAD^+^ to NADH and NADP^+^ to NADPH

**Fig. 3:**
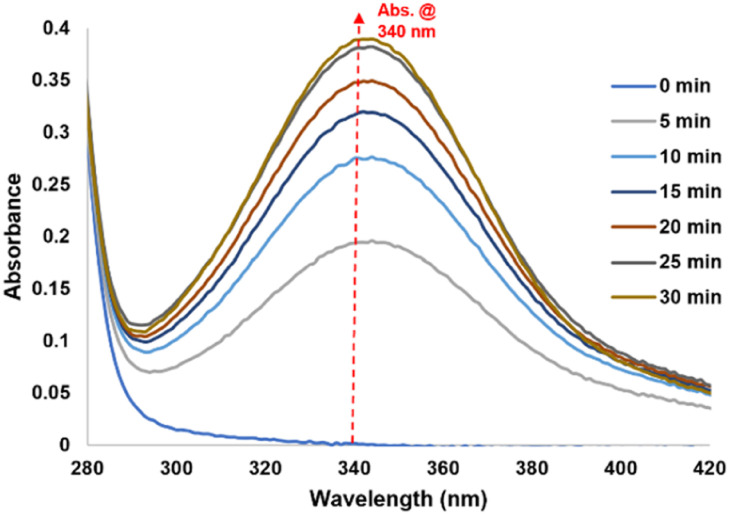
Reaction kinetics of 2 mM NAD^+^ with CORM-A1 (1 : 1, 2 mM) incubated at 37 °C for 30 minutes monitored using UV-Vis.

First, the reaction of CORM-A1 and NAD^+^ was analyzed using UV-Vis by taking advantage of the lack of absorbance for NAD^+^ at 340 nm, where there is an intense peak for NADH.^43^ We conducted the experiments at μM to low mM concentrations, since cellular NAD^+^ concentrations range between approximately 0.2–0.5 mM.^44^ Specifically, when NAD^+^ and CORM-A1 (2 mM each) were incubated in 10 mM PBS at 37 °C for 15 minutes, time-dependent increase of the peak corresponding to NADH (340 nm) was observed. Fig. 3 shows a representative set of UV spectra. Further, the observed spectral changes are also dependent on the concentration and relative ratio between CORM-A1 and NAD^+^. In order to quantitatively assess the yield of NADH, a standard curve of absorptions of NADH (*λ*_max_ = 340 nm) was established (Fig. S1A†). For the reaction between 2 mM NAD^+^ with 2 mM CORM-A1, 1.4 mM NADH was formed at the 15 min point (70%, marked by a red dash line) and 1.75 mM (88%) was formed at the 30 minute point (Fig. 3).

**Fig. 4:**
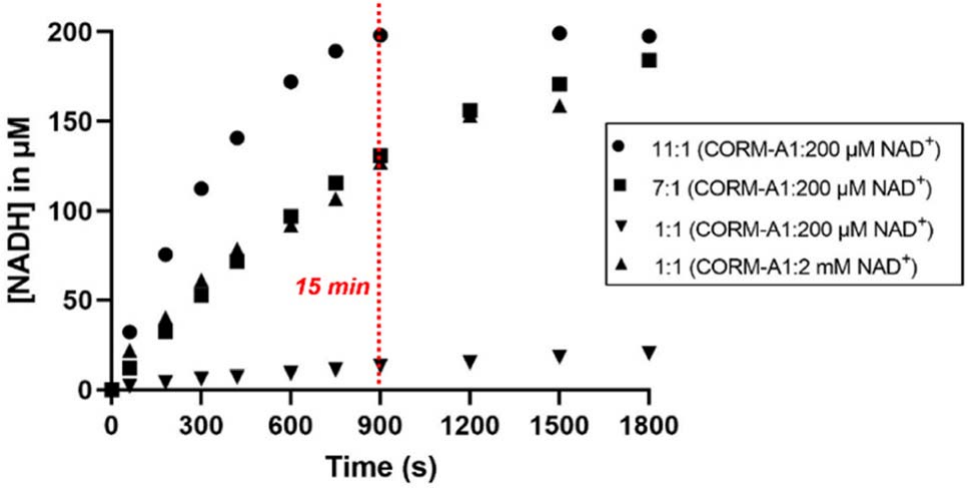
Kinetic studies of reaction of NAD^+^ with different concentrations of CORM-A1 (1 : 1, 2 mM or 200 μM; 7 : 1 1.4 mM; 11 : 1 2.2 mM). The 1 : 1 reaction (2 mM CORM-A1: 2 mM NAD^+^) was incubated at in PBS at 37 °C. At each time point, the reaction was either conducted at or diluted by 10-fold to 200 μM for UV measurements. Other reactions were conducted at 200 μM (NAD^+^) for UV-Vis detection. Reactions were monitored using UV-Vis at 340 nm.

When CORM-A1 was used in excess (11 : 1, 2.2 mM CORM-A1: 200 μM NAD^+^), about 200 μM NADH was formed after 15 min (Fig. 4), indicating 100% completion (Fig. 4). These results confirmed the ability for CORM-A1 to chemically reduce NAD^+^ in an aqueous solution. When an equal molar ratio was used at 200 μM CORM-A1, a significant decrease in the rate was observed in comparison to the reaction at 2 mM CORM-A1. These expected concentration-dependent changes in reaction rate are consistent with the biomolecular nature of the reaction. Consequently, only when CORM-A1 was in a ≥10-fold molar ratio, complete reaction was observed within 15 minutes.

**Fig. 5:**
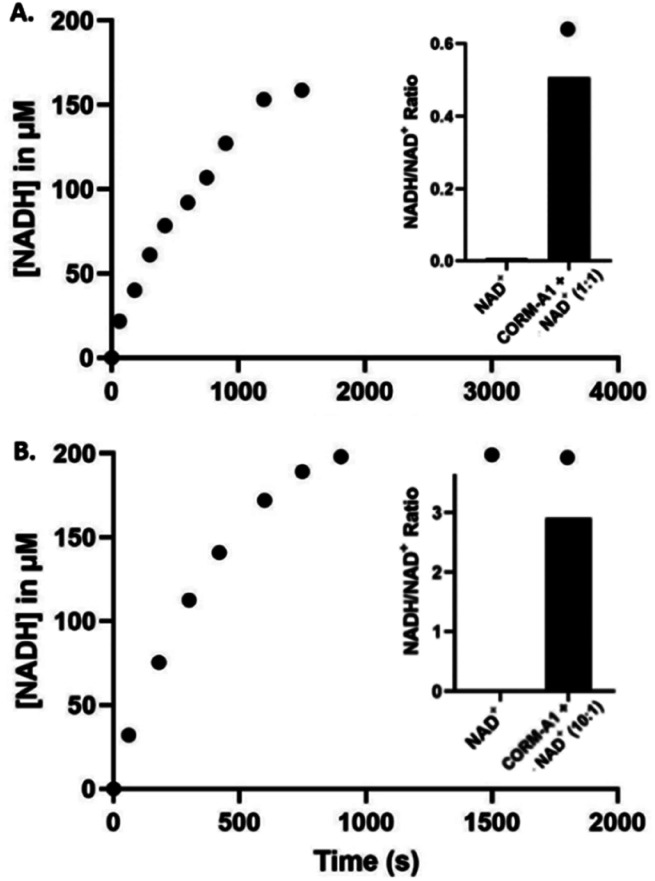
(A) Reaction of 2 mM NAD^+^ with CORM-A1 (1 : 1, 2 mM) incubated at 37 °C for 30 minutes. Reactions monitored and AUC determined using LC-MS/MS (right) and UV-Vis (left). (B) Reaction of 1 mM NAD^+^ with CORM-A1 (10 : 1, 10 mM) incubated at 37 °C for 15 minutes. Formation of NADH (μM) over 30 minutes by reaction of 200 μM NAD^+^ and CORM-A1 (11 : 1, 2.2 mM) monitored by LC-MS/MS (right) and UV-Vis (left), quantification done using AUC. Ratios are calculated using the average AUC from duplication experiments.

The conversion of NAD^+^ to NADH was further confirmed with LC-MS/MS (Fig. S4–S18†). Specifically, LC-MS/MS results indicated complete reduction of NAD^+^ to NADH within 15 minutes at 10 : 1 CORM-A1 : NAD^+^ ratio and 30 minutes at 1 : 1 CORM-A1 : NAD^+^ ratio, corroborating findings of the UV-Vis experiments. When reactions were conducted at 37 °C in unbuffered water (pH 5.5), LC-MS/MS studies (Fig. S4–S18†) confirmed NADH formation and showed an increase in NADH/NAD^+^ ratio. Using an equivalent molar ratio (1 : 1) of CORM-A1 and NAD^+^ at 2 mM each, a 65-fold change in NADH/NAD^+^ ratio was observed within 30 minutes (Fig. 5A). When the molar ratio of CORM-A1 : NAD^+^ was increased to 10 : 1, NAD^+^ was completely consumed within 15 minute and NADH/NAD^+^ ratio increased by 260-fold (Fig. 5B). These results are consistent with observations in the UV-Vis experiments and confirm the ability for CORM-A1 to quantitatively reduce NAD^+^ to NADH in a pure chemical reaction.

**Fig. 6:**
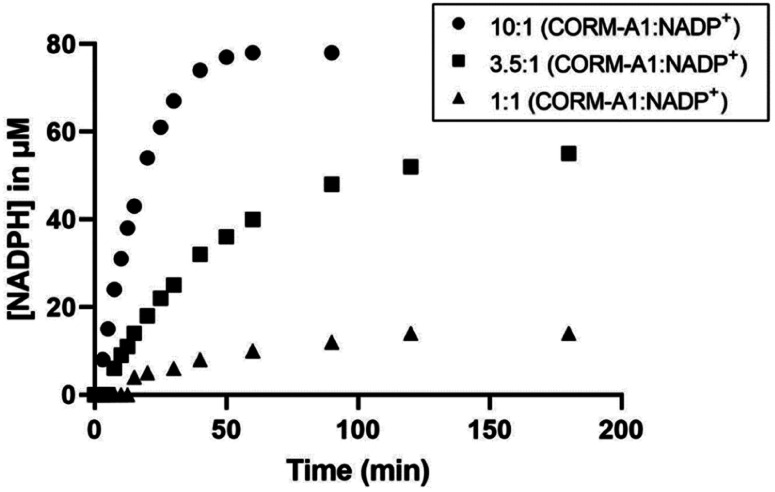
Kinetic studies of reaction of NADP^+^ with CORM-A1 at different concentrations and ratios (1 : 1, 100 μM; 3.5 : 1 350 μM; 10 : 1 1 mM). Reactions were conducted at 100 μM NADP^+^. NADPH generation was monitored by UV-Vis absorbance at 340 nm.

Similar studies were conducted to evaluate the reaction between NADP^+^ and CORM-A1. It was found that CORM-A1 was able to reduce NADP^+^ to NADPH, in a similar fashion as the reduction of NAD^+^. In one example, reaction between 1 mM CORM-A1 and 100 μM NADP^+^ in PBS at 37 °C (Fig. 6) led to the formation of 78 μM of NADPH within 60 minutes. At the same time point, lower concentrations of CORM-A1 at 350 μM and 100 μM led to the formation of 40 μM and 10 μM of NADPH, respectively. Combined, the results presented suggest that CORM-A1 has the chemical reactivity to play a significant role in affecting the concentrations of two biological cofactors (NAD^+^ and NADP^+^), which are important for biochemical processes such as glycolysis and the pentose phosphate pathway.

We also conducted ^11^B-NMR experiments to monitor the consumption of the BH_3_ moiety in CORM-A1 by NAD^+^ in D_2_O. In the presence of 33.3 mM NAD^+^, a significant decrease in intensity of the borane peaks was seen within 10 minutes. When the same reaction was tested at 30 minutes, no further change in peak intensity was observed, indicating fast reaction. When an additional 66.6 mM of NAD^+^ was added to the reaction, giving a final reaction of 1 : 1 CORM-A1 : NAD^+^, the borane peaks disappeared within 10 minutes. These results further support a direct reaction between NAD^+^ and the borane moiety on CORM-A1 (Fig. S19†).

The chemical reactivities of CORM-A1 observed are in general agreement with basic organic chemistry and the known reactivity of borohydride and borane reagents such as cyanoborohydride. This newly observed chemical reactivity of CORM-A1 needs to be taken into consideration when CORM-A1 is used for studying the effect of CO. It should be noted that there have been some specific examples, in which NAD(P)^+^/NAD(P)H were reported to be involved in the biological functions of CO. In one set of studies, the platelet inhibition effects of CORM-A1 were proposed to be through conversion of NAD^+^ to NADH.^28,29^ In another study, the role of CORM-A1 was proposed to regulate the concentrations of biological co-factors, such as NADP^+^. For CORM-A1's ability to improve neuronal differentiation through increasing GSH levels, an earlier report suggested the pathway involving enhanced generation of NADPH.^40^ The newly established chemical reactivity of CORM-A1 toward NADP^+^ and NAD^+^ suggests the likely need to re-evaluate the functions of CO and its mechanism(s) of action by taking into consideration of the redox properties of CORM-A1. However, it is understood that the cellular environment is much more complex than solution-phase chemistry. Therefore, factors beyond the reactivity of CORM-A1 in solution-phase may need to be considered in any re-evaluation efforts. In any case, there is a high degree of certainty that the chemical reactivity of CORM-A1 is a strong confounding factor to consider in result interpretations.

### Criterion II. Assessment of commonly used negative controls: iCORM-A1

#### iCORM-A1 does not serve as an adequate negative control for the reducing ability of CORM-A1

**Fig. 7:**
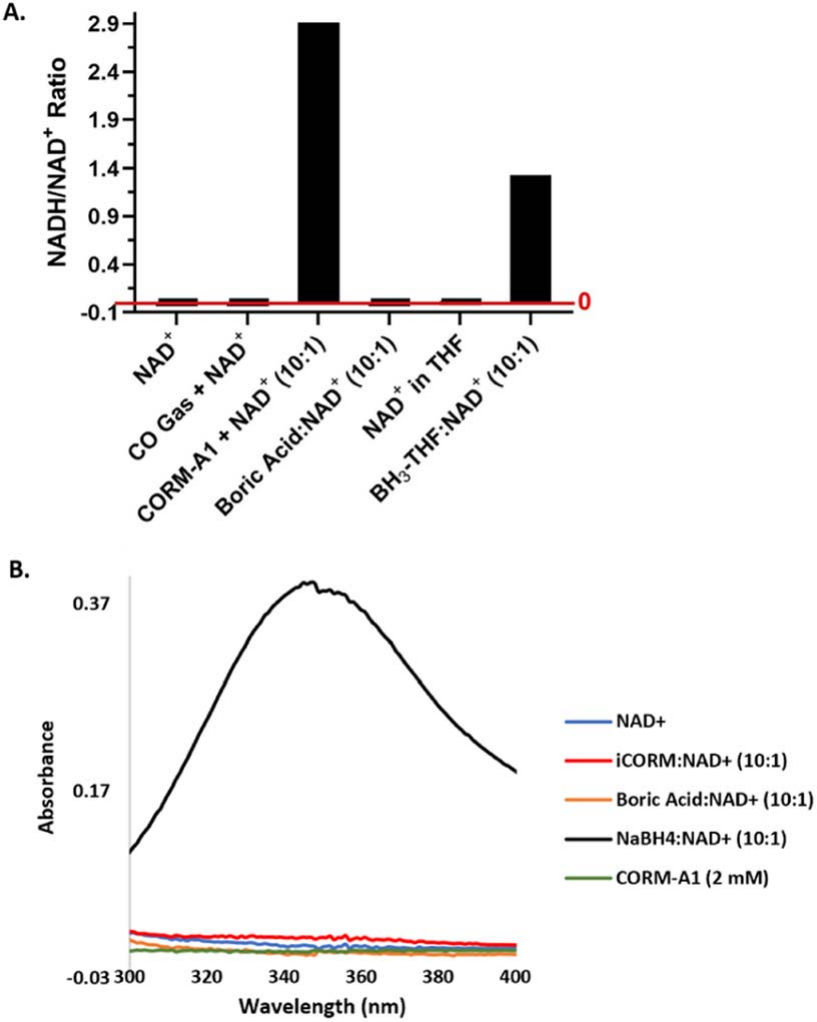
Reactions of NAD^+^ with CO gas, CORM-A1 and other relevant boron species. (A) Reactions of 1 mM NAD^+^ with CO gas (2 mL), CORM-A1 (10 : 1, 10 mM), boric acid (10 : 1, 10 mM), or BH_3_-THF (10 : 1, 10 mM) at 37 °C for 15 minutes. Reactions monitored and AUC determined using LC-MS/MS. Ratios are calculated using the average AUC from duplication experiments. (B) Reaction of 1 mM NAD^+^ with iCORM-A1 (10 : 1, 10 mM), boric acid (10 : 1, 10 mM) and NaBH_4_ (10 : 1, 10 mM). Unreacted CORM-A1 (2 mM) provided as a control. Reactions were diluted to 200 μM after 15 minutes for UV-vis measurements.

In addressing the implications of the newly established chemical reactivity of CORM-A1 in its use in studying CO biology, one key question is whether there are adequate negative controls to allow for examination of only CO-dependent effects. One commonly used “negative control” is “CO-depleted” CORM-A1 (inactivated CORM or iCORM). In this context, we would like to note that it is nearly impossible to have a “perfect” negative control of a prodrug of almost any active agent. Thus, when we look at the “negative control” issue, we bear this in mind and only examine truly significant issues that have a high likelihood of significantly complicating result interpretation, not mere minor or remote possibilities. As such, there are many ways of looking at this issue. In this study, we only examine the negative control issue in the context of chemical reactivity, specifically, the chemical reactivity of the iCORM preparations towards NAD(P)^+^. It should be noted that several recent publications have already revealed complications in using various iCORMs to properly control for the reactivity of the donor itself.^6,26,31,33,45^ Typically, iCORM-A1 is prepared by adding 0.1 M HCl to a stock solution of CORM-A1 and then bubbling the solution with N_2_ gas to remove any residual CO. In doing so, it is assumed that CORM-A1 is converted to sodium borate and/or boric acid in equilibrium, depending on the pH.^26^ This assumption is certainly in agreement with what one would expect based on borane chemistry.^46^ The borane moiety present in the structure of CORM-A1, as well as the previously published mechanism of CO release, permits the assumption that the by-product is boric acid, given that borane, the only remaining component after CO release, produces boric acid and hydrogen gas when in aqueous solution.^47^ The production of boric acid from borane in aqueous solution is an intrinsic redox reaction. Based on the CO release mechanism in Scheme 1, it is reasonable that the transformation from CORM-A1 to iCORM-A1 is analogous to this redox reaction. Therefore, we studied the reactivity of the same iCORM-A1 preparation, with NAD^+^ using UV-Vis and found no indication that iCORM-A1 was able to reduce NAD^+^ in the same fashion as CORM-A1 (Fig. 7B).^26^ Agreeing with UV-Vis data of iCORM-A1, LC-MS and UV-Vis studies also showed that boric acid had no effect on NAD^+^ depletion (Fig. 7A and B). Overall, it is clear that CORM-A1 participates in a CO-independent redox reaction with NAD^+^, which cannot be achieved with iCORM-A1 or boric acid. Therefore, these results suggest that iCORM-A1 and boric acid are not proper negative controls for the redox properties of CORM-A1 in its use as a CO donor, at least in the context of their chemical reactivity.

#### Other controls for CORM-A1

##### CO gas

Chemically, one would unambiguously expect CO to be inert toward NAD^+^. For the sake of thoroughness, we further confirmed this aspect using data. Thus, 1 mM NAD^+^ was incubated with an excess amount of pure CO gas (2 mL, approximately 90 μmol of CO) in a 1 mL HPLC vial at 37 °C for 15 minutes. LC-MS studies of this reaction mixture indicated no change to this solution that could suggest a reduction of NAD^+^ (Fig. 7A), as one would expect.

##### Borane-based compounds

The direct chemical reduction of NAD^+^ and NADP^+^ by CORM-A1, along with the lack of redox reactivity seen with iCORM-A1 and boric acid, suggests the complexed borane being the reducing agent. As discussed earlier, borane is a textbook case of a strong reducing agent, especially when complexed with a Lewis base.^48,49^ Examples include BH_3_-ether, BH_3_-THF, and sodium cyanoborohydride, which is known to reduce NAD^+^.^41,42,50^ For comparison, we conducted additional studies of NAD^+^ reduction by NaBH_4_ and BH_3_-THF using UV-Vis and LC-MS, respectively. The reaction between BH_3_-THF and NAD^+^ (10 : 1) in 5% water in THF solution similarly led to a significantly reduced level of NAD^+^ with the concomitant formation of NADH. At the 15 min point, the ratio of NADH/NAD^+^ increased to 1.33 from the initial 0.026 (Fig. 7A). Using UV-Vis, the reaction between NaBH_4_ and CORM-A1 was probed. When incubated for 15 minutes at a 10 : 1 molar ratio, 10 mM NaBH_4_ led to the formation of 440 μM NADH from 1 mM NAD^+^, suggesting a weaker reducing ability as compared to CORM-A1 (Fig. 7B). The decreased level of reduction may be due to a slower reaction rate or increased hydrolysis of NaBH_4_. Collectively, these results are consistent with the borane moiety on CORM-A1 being a strong reducing agent, analogous to other BH_3_ complexes. In summary, CORM-A1 has redox activity that is independent of gaseous CO and is not properly controlled with iCORM-A1 or boric acid (Fig. 2).

### Criterion III: the ability for CORM-A1 to generate CO in a reproducible and reliable fashion: CO production under various conditions

The entire premise of using CORM-A1 as a CO donor or surrogate for studying CO biology is predicated on its presumed ability to donate an adequate amount of CO in a reproducible and controllable fashion. Along this line, there have been studies of CO release yield and kinetics from CORM-A1 using the myoglobin assay. In doing so, sodium dithionite is used to keep myoglobin in the reduced form, which scavenges CO in solution, leading to spectroscopic changes. Using such a method, CORM-A1 was reported to show pH- and temperature-dependent CO release, with a half-life of 2.5 minutes and 21 minutes at 37 °C when incubated at pH 5.5 and pH 7.4, respectively.^26^ Given the redox activity of CORM-A1 (described above), we were interested in using an GC quantification method to examine CO release to avoid introducing additional factors in CO detection. Specifically, without the need to add sodium dithionite (used in the myoglobin assay), the possible chemical interactions between two redox-active species in dithionite and CORM-A1 is eliminated. It should be noted that Klein and co-workers reported pH-dependent CO release of up to 0.91 ± 0.09 mole of CO liberated per mole of CORM-A1 using gas-phase vibrational spectroscopy.^51^ When incubated at 27.5 °C and pH 7.4, Klein and co-workers determined the half-life of CORM-A1 being 106 minutes for the formation of the borane carbonyl intermediate, suggesting an overall half-life of about 2 hours. The authors suggested this half-life as being comparable to previous reports due to the slight difference in pH and temperature.

Aimed at probing the effect of temperature and pH, we incubated CORM-A1 in 100 mM PBS at both 37 °C and room temperature and did not observe significant changes in the rate of CO production. At 37 °C, the CO yield was 15% in the first 15 minutes and 60% within 24 hours (Table 2, entry 3). In contrast, 13% CO was generated in the first 15 minutes and 54% within 24 hours at room temperature (Table S1,† entry S1). These results, along with the results reported by Klein and co-workers, suggest that CO production from CORM-A1 does not have the kind of temperature-dependence to account for the reaction half-life difference between the original report and that of Klein and co-workers. Although FTIR of the gas headspace removes the complications of additional reagents (*i.e.*, dithionite), this is an indirect method of quantification of CO involving a fit line calculated from a sum of CO concentration and intermediate H_3_BCO concentration.^51^ Given the various numbers in the literature under different conditions, we feel the need to use the gold-standard method, GC, to conclusively assess CO release from CORM-A1. Therefore, we conducted quantitative analysis of CO release of CORM-A1 under near physiological condition in both unbuffered water (pH = 5.5) and PBS (pH = 7.4) by using a methanizer coupled GC-FID, which is much more sensitive than the GC-TCD method and ensures the detection of CO at sub-ppm level.

In examining the ability for CORM-A1 to release CO, there are aspects that need to be characterized. First, we are interested in studying CO release from CORM-A1 in water, as it has been reported to be stable in water, as well as the effect of buffer.^27^ We are also interested in the pH effect on CO release in pure water, as the somewhat basic nature of the CORM-A1 was stated to be the reason for its stability in water. Second, we are interested in how CORM-A1's reducing capability of NAD^+^ and NADP^+^ may affect CO production. Third, we are interested in probing other factors that could play a role in affecting CO release from CORM-A1, including its redox activity, the presence of organic and inorganic ions, oxidants, and commonly used reagents and media commonly used in experiments to study CO's biology.

### Criterion III, Assessment 1. CO production in unbuffered water and buffered solutions

#### CO production in water and pH effect

CORM-A1 was reported to be stable in water due to its basic nature, ultimately changing the pH of the solution to around pH 11.^27^ To our knowledge, there are no concentration information reported or in-depth stability studies of CORM-A1 in aqueous solution. As the conjugate base of a weak acid, we expect the pH of the solution to be dependent on the concentration of CORM-A1. Therefore, we prepared CORM-A1 solutions at 20 μM, 100 μM, 500 μM, 1 mM, 10 mM, and 100 mM. Indeed, the pH values of these solutions were determined to be 5.5, 6.0, 6.5, 7.5, 9.0 and 9.5, respectively. As expected, the pH depends on the concentration of CORM-A1; such results are different from the common belief that CORM-A1 solution is basic. Apparently, CORM-A1 is a weak base and can bring the pH to 9.5 only at a high concentration. At low concentrations (*e.g.*, 20 μM), the pH remains about the same as the water used (pH 5.5). Further, these solutions of CORM-A1 in unbuffered water showed initial CO release of ≤2% at the 15 min point regardless of the final pH (Table 1). Especially surprising was the low CO yield of the solution at pH 5.5, which was reported to allow for CO release with a *t*_1/2_ of 2.5 min in buffer with a similarly adjusted pH.^26^ Such results suggest that low pH (*e.g.*, 5.5) does not automatically lead to rapid CO release and CORM-A1 stability in aqueous solution is not exclusively pH-dependent.

**Table 1:** CO production of CORM-A1 in unbuffered water (*n* = 3)

Entry	[CORM-A1]	Initial CO yield% (15 min)	Total CO yield% (20+ h)	Final pH
1	20 μM	1 ± 0.4	24 ± 6.5	5.5
2	100 μM	2 ± 1.4	24 ± 7.3	6.0
3	500 μM	0.7 ± 0.2	24 ± 3.6	6.5
4	1 mM	0.5 ± 0.2	25 ± 4.0	7.5
5	10 mM	0.6 ± 0.2	33 ± 2.4	9.0
6	100 mM	0.4 ± 0.2	23 ± 3.5	9.5

#### Effect of buffer

As previously discussed, CORM-A1 was reported to have a rapid CO release at low pH, something we did not observe in unbuffered water solutions. The sole difference between the data in Table 1 and the high CO release yield at low pH (5.5) reported by the literature was the use of buffer. Specifically, CORM-A1 was reported to release CO in buffered aqueous conditions such as PBS with a *t*_1/2_ of 2.5 min at pH 5.5. Considering the low CO production yield in water regardless of pH (Table 1), we became interested in examining the effect of buffer by using PBS at different buffer strengths and CORM-A1 concentrations, respectively. First, different concentrations of CORM-A1 were tested in 100 mM PBS solution at 37 °C. In 100 mM PBS (pH 7.4), CORM-A1 at 10 mM was found to release CO with a 15% yield at the 15 min point (hereafter denoted as initial period) and a total of 60% after 20+ hours (entry 3, Table 2). At 1 mM of CORM-A1, an average of 13% CO was released within the first 15 minutes and 71% overall CO yield after 20 h. At 100 μM of CORM-A1, CO release of 3% and 45% was seen for the initial period and after 20 h, respectively (entries 1 and 2, Table 2). These numbers are much higher than what was observed in the absence of a buffer. To further probe the instability of CORM-A1 in buffered aqueous solutions, CO release from CORM-A1 in different concentrations of PBS was tested. Commercially available PBS solutions of 1× (10 mM), 10× (100 mM), and 20× (200 mM) were used. As can be seen in Table 2, at 10 mM of PBS (1×), the initial CO yield from 10 mM CORM-A1 was only 1%, followed by a total CO yield of 48% (entry 4, Table 2). Higher concentrations of PBS (100 mM and 200 mM) showed initial CO yields of 15% and 18%, respectively (entry 3 and 5, Table 2). However, the total CO yields after 20 h are in the range of 45–71% with lower yields observed at either a low CORM-A1 concentration (*e.g.*, 100 μM) or a low buffer concentration (*e.g.*, 10 mM). The observed dependence of CO yield for the initial period on the concentration of the buffer and CORM-A1 suggests a bimolecular event involving the buffer components. The low yields and release rate from the experiments in water further support this notion. Overall, the initial CO release yield is low. Further, the overall results are substantially different from the literature report of *t*_1/2_ of 21 min in PBS at neutral pH. Again, it is important to note that a reducing agent (sodium dithionite) was used in the experiments using the literature-described myoglobin assay. Sodium dithionite has been previously reported to interact with other CORMs to promote CO production. Thus, the conditions we used by assessing gas production directly are different from that of the original report *via* an indirect method for measuring gas production using myoglobin in the presence of dithionite.

**Table 2:** Effect of buffer on CO production from CORM-A1 (*n* = 3)

Entry	[CORM-A1]	Solvent	Initial CO yield% (15 min)	Total CO yield% (20+ h)
1	0.1 mM	100 mM PBS	3.0 ± 0.7	45 ± 10.5
2	1 mM	100 mM PBS	13 ± 5.0	71 ± 1.9
3	10 mM	100 mM PBS	15 ± 5.5	60 ± 3.6
4	10 mM	10 mM PBS	1.0 ± 0.15	48 ± 5
5	10 mM	200 mM PBS	18 ± 2	57 ± 10.3

### Criterion III, Assessment 2. The effect of CORM-A1's redox activity with NAD^+^ and NADP^+^ on CO production

#### NAD^+^ and NADP^+^ accelerate CO release from CORM-A1 in PBS

Solutions containing an equal molar ratio of NAD^+^ showed a higher initial CO yield in 100 mM PBS by about two-fold, 35% at 10 mM and 21% at 1 mM within 15 minutes (entry 2, Table 3 and entry 4, Table 4) than in the absence of NAD^+^. To further examine this issue, we also studied the effects of excess NAD^+^. Specifically, when 10 mM CORM-A1 was incubated with 30 mM NAD^+^ in 100 mM PBS solution, initial CO yield of 55% was obtained (entry 3, Table 3). Obviously, the presence of 3-fold excess of NAD^+^ led to an approximately 3.5-fold increase in the initial CO yield over the reaction in solely PBS. However, the overall CO yield (20+ h) was comparable to solutions that did not contain NAD^+^. In a similar fashion to NAD^+^, equal molar equivalents of NADP^+^ increased the CO release yield from 10 mM CORM-A1 with an initial CO yield of 49% and an overall CO yield of 58% (entry 4, Table 3).

**Table 3:** Effect of different reagents on the CO production from 10 mM CORM-A1 in 100 mM PBS (*n* = 3)

Entry	Added reagent	Initial CO yield% (15 min)	Total CO yield% (20+ h)
1	—	15 ± 5.5	60 ± 3.6
2	10 mM NAD^+^	35 ± 9.8	64 ± 4
3	30 mM NAD^+^	55 ± 5.7	60 ± 8.3
4	10 mM NADP^+^	49 ± 2.9	58 ± 2.7

**Table 4:** Effect of NAD^+^ on the stability of 1 mM CORM-A1 in unbuffered water (*n* = 3)

Entry	Solvent	Added reagent	Initial CO yield% (15 min)	Total CO yield% (20+ h)
1	Unbuffered water	—	0.5 ± 0.2	25 ± 4.0
2	100 mM PBS	—	13 ± 5.0	71 ± 1.9
3	Unbuffered water	1 mM NAD^+^	8 ± 2.0	47 ± 8.3
4	100 mM PBS	1 mM NAD^+^	21 ± 7.8	54 ± 4

#### NAD^+^ reverses CORM-A1's stability in water

As previously discussed, CORM-A1 is kinetically more stable in unbuffered water solutions than in PBS. With the demonstrated acceleration effect of NAD^+^ on CO release in PBS, we became also interested in studying the effect of NAD^+^ on the stability of CORM-A1 in unbuffered water and thus CO release from CORM-A1. The addition of NAD^+^ had the same general effect in water as in PBS. For example, NAD^+^ at 1 mM increased the initial CO release from CORM-A1 (1 mM) by 16-fold from 0.5% to 8% (entry 1 and 3, Table 4). Such results highlight the interesting ability of NAD^+^ to trigger CO release from CORM-A1 at a rate comparable to that in buffered solution. Such results further suggest that CO release from CORM-A1 is not solely pH-dependent. More mechanistic studies need to be done to probe the stability of CORM-A1 in aqueous solution.

### Criterion III, Assessment 3. Probing other factors that impact CO production from CORM-A1: the idiosyncratic nature of CO production

In an attempt to define a clear mechanism for CO production from CORM-A1 to account for the above observations, many different factors were probed. These factors were selected based on previously reported studies using CORM-A1 or relevance to the literature studies that give us reasons to examine their effects. Some of these factors include the effect of ions, the redox activity of CORM-A1, the presence of different oxidants including H_2_O_2_, DMSO, DDQ, and sGC inhibitor, ODQ. We also examined CO production from CORM-A1, or lack thereof, in biological media. The studies discussed hereon reveal that the mechanism of CO release from CORM-A1 is difficult to define, following a seemingly idiosyncratic pattern. Along the same lines, these studies bring to light complications in studies using CORM-A1 as a CO surrogate to study CO's biology.

#### Effect of ions

**Fig. 8:**
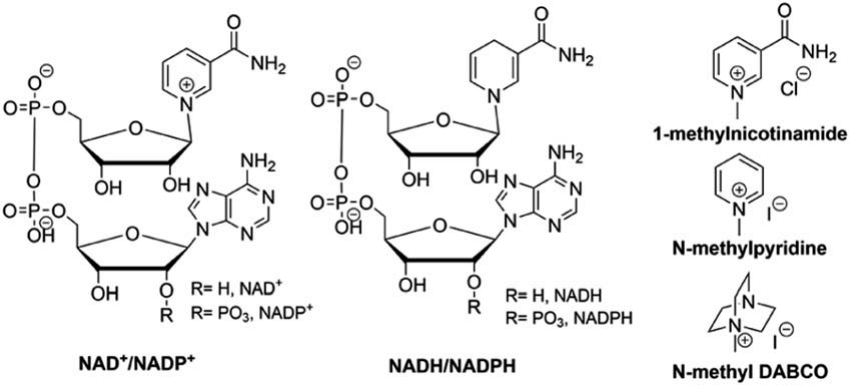
Structures of NAD^+^/NADP^+^, NADH/NADPH, 1-methylnicotinamide, *N*-methylpyridine, and *N*-methyl DABCO.

To examine the effects of various ions, other solutions containing similar anions and cations were tested. First, the CO production from 10 mM CORM-A1 in the presence of 100 mM NaCl was tested to see whether it was the sodium cation in PBS that led to the rate enhancement. In the presence of NaCl in unbuffered water at pH 5.5, CORM-A1 produced negligible amounts of CO, 0.4% in the first 15 minutes, and 33% over the course of 20 hours (entry 4, Table 5). These results are comparable to the CO production of CORM-A1 in unbuffered water solutions, suggesting no significant role for sodium cations (or chloride anions) in CO release from CORM-A1. Such results further suggest the important roles of phosphate, but not chloride. Second, the role of inorganic cations was further probed by testing CO production from 10 mM CORM-A1 in two concentrations of lithium phosphate buffer (25 mM and 100 mM) at pH 7.4. Both concentrations produced comparable initial CO yields of 16% and 18%, respectively, within 15 minutes (entry 1 and 3, Table 5). These results are similar to the CO production from CORM-A1 in PBS, further suggesting the lack of a direct role for inorganic cations. On the other hand, the presence of phosphate anions seems beneficial to the production of CO from CORM-A1. Along the same line, in the presence of NAD^+^, the CO yield from 10 mM CORM-A1 in 25 mM lithium phosphate buffer was 51% in the first 15 minutes, a significant increase in comparison to PBS solutions containing the same concentrations of CORM-A1 and NAD^+^ (entry 2, Table 5). Because NAD^+^ also has a pyrophosphate group (Fig. 8), we cannot readily conclude whether the CO release rate was due to the cation, the anion, the redox reaction, or a combination of three. Due to CORM-A1's ability to reduce the nicotinamide moiety (an organic cation), additional organic cations were tested (Fig. 8). Neither the presence of 10 mM methylated pyridine nor 10 mM methylated DABCO in 100 mM PBS enhanced the CO production from 10 mM CORM-A1, giving initial CO yields of 12% and 15% in 15 minutes, respectively (entries 5 and 6, Table 5). These results suggest that NAD^+^ being an organic cation is unlikely to be what triggers the accelerated CO release. Then, we reasoned that the role of NAD^+^ is likely through its reduction by CORM-A1, as further discussed in the next section. In summary, organic anions, but not organic or inorganic cations seem to play a role in the CO production from CORM-A1.

**Table 5:** Effect of different ions on CO production from 10 mM CORM-A1 (*n* = 3)

Entry	Solvent	Added reagent	Initial CO yield% (15 min)	Total CO yield% (20+ h)
1	25 mM lithium phosphate buffer	—	16 ± 1.1	51 ± 12.4
2	25 mM lithium phosphate buffer	10 mM NAD^+^	51 ± 5.5	68 ± 7.5
3	100 mM lithium phosphate buffer	—	18 ± 0.7	55 ± 1.1
4	100 mM NaCl	—	0.4 ± 0.0	33 ± 7.1
5	100 mM PBS	10 mM *N*-methylpyridine	12 ± 4.9	47 ± 4
6	100 mM PBS	10 mM *N*-methyl DABCO	15 ± 1.2	47 ± 5.7

#### Redox activity promotes CO production

To broaden the chemical reactivity studies, we also examined the reaction with the *N*-methylnicotinamide moiety (Fig. 8), which is present in both NAD^+^ and NADP^+^ and is the redox center of these two co-factors. Specifically, reaction between 10 mM 1-methylnicotinamide and 10 mM CORM-A1 led to an initial CO release yield of 32% (entry 2, Table 6). Similar to NAD^+^ and NADP^+^, CORM-A1 reduced 1-methylnicotinamide in the same manner as NaBH_4_, further supporting the reduction of the nicotinamide moiety on NAD^+^ and NADP^+^ by CORM-A1 being responsible for its acceleration in CO release (Fig. S3†). The role of CORM-A1's reducing capability in accelerating CO release was investigated using NADH, which is already reduced. The addition of NADH (10 mM) to CORM-A1 (10 mM) led to a 13% yield of CO release in the first 15 min, which is comparable to buffer alone (entry 1, Table 6). These results suggest that the reduction of the methylated nicotinamide moiety on NAD^+^ and NADP^+^ by CORM-A1 facilitates its CO release. Since methylated pyridine did not enhance CO production, it is possible that the electron-withdrawing nature of the amide group on nicotinamide could play a role in the ease of its reduction, which facilitates CO release. Further, borane has long been shown to coordinate to carbonyl oxygens to promote reduction reactions. Therefore, these results also suggest the possibility of coordination of a boron species to the carbonyl oxygen on the nicotinamide of NAD^+^, leading to accelerated NAD^+^ reduction to NADH and CO release.^50^ Further the phosphate group of NAD(P)^+^ might also play a role in the same way as phosphate buffer.

**Table 6:** Effect of nicotinamide variations on the CO production from 10 mM CORM-A1 in 100 mM PBS (*n* = 3)

Entry	Added reagent	Initial CO yield% (15 min)	Total CO yield% (20+ h)
1	10 mM NADH	13 ± 5.3	49 ± 3.9
2	10 mM 1-methylnicotinamide	32 ± 8.8	51 ± 9

#### Oxidants alter CO production

The ability to accelerate CO release from CORM-A1 through reduction of NAD^+^ begs the question as to whether oxidation of the BH_3_ moiety would lead to CO release. Therefore, the effect of a few different types of relevant oxidants were examined. This section discusses this and a few other factors.

##### DMSO inhibits CO production from CORM-A1

First, CO production from CORM-A1 in DMSO, an oxidant and commonly used solvent, was absent. At first, it seemed that CORM-A1 was stable in DMSO, but further experiments showed that stock solutions of CORM-A1 in DMSO failed to produce CO when diluted in 100 mM PBS (entry 1, Table 7). We recognize that biological studies often use a very small amounts of DMSO in PBS, with an acceptable level often being about 0.1–0.5% DMSO. Therefore, CO release from CORM-A1 in 0.5%DMSO/PBS solutions was also probed (entry 2, Table 7). CO production from 1 mM CORM-A1 in 0.5% DMSO/PBS solutions was diminished in comparison to 1 mM CORM-A1 in just PBS solutions (10 mM) with an initial CO yield of 2% and total CO yield of 42%. These results suggest that CORM-A1 has some chemical reactivities with DMSO that diminishes or completely inhibits its CO production. Surprisingly, when a different organic oxidant, DDQ, was included in DMSO-only solutions, this inhibition was slightly ameliorated, increasing the CO release from 0% to 18% in the first 15 minutes and 2% to 26% within 24 hours (entry 3, Table 7).

**Table 7:** CO production from CORM-A1 in the presence of different DMSO solutions and oxidants (*n* = 3)

Entry	[CORM-A1]	Solvent	Added reagent (oxidant)	Initial CO yield% (15 min)	Total CO yield% (20+ h)
1	10 mM	DMSO	—	0 ± 0.1	2 ± 1.0
2	1 mM	0.5%DMSO/PBS	—	2 ± 0.4	42 ± 2.6
3	10 mM	DMSO	10 mM DDQ	18 ± 4.5	26 ± 3.3
4	100 μM	DMSO	10 μM ODQ	14 ± 5.5	20 ± 4.3

##### sGC inhibitor, ODQ, alters CO production from CORM-A1

The therapeutic role of CO gas is often studied in the context of its ability to activate soluble guanylate cyclase (sGC).^52^ To such an effect, many of the biological studies using CORMs also include studies with a sGC inhibitor, ODQ.^29,53,54^ ODQ is not soluble in aqueous solutions and must be administered using a mixed solvent of DMSO/PBS. CORM-A1 is readily soluble in aqueous solution, so its CO production in DMSO has not yet been discussed in literature to our knowledge. When 100 μM CORM-A1 was incubated with 10 μM ODQ in DMSO, significantly increased CO production was observed in the first 15 minutes (14%) as compared to unbuffered water and in DMSO. Such results are similar to the addition of DDQ as described earlier. However, the overall production at 24 hour-point was still only 20% (entry 4, Table 7). Although DMSO was not used as a solvent for CORM-A1, the change in CO production in the presence of ODQ suggests chemical reactivity between ODQ and CORM-A1. The mechanistic aspects of the reaction need to be further investigated, but ultimately the results suggest that the presence of CORM-A1 may impact biological results involving ODQ. Furthermore, these results convolute the role of oxidants in the CO release mechanism of CORM-A1.

##### H_2_O_2_ diminishes CO release from CORM-A1

The unclear role of oxidants brings concerns in the interaction between commonly used redox reagents in CORM-A1 experiments studying CO's biology. Therefore, we probed CO production from CORM-A1 in the presence of H_2_O_2_, a commonly used reagent to induce oxidative stress. Incidentally, H_2_O_2_ is also well-established in textbook hydroboration reactions to oxidizes the –BH_2_ moiety, after its reduction of an alkene. Our lab has previously reported that 100 μM CORM-A1 degrades 300 μM H_2_O_2_ in a non-catalytic manner.^30^ Due to CORM-A1's ability to degrade H_2_O_2_ in a non-catalytic manner, one can assume a stoichiometric redox process involving relevant structural moiety of CORM-A1, similar to its reaction with NAD^+^. The previously reported reaction was further investigated in the context of CO release in H_2_O_2_/PBS solutions. In the presence of 300 μM H_2_O_2,_ 100 μM of CORM-A1 had an initial CO yield of <1% and a total CO yield of 2% after 20 h (entry 3, Table 8). In the presence of lower concentrations of H_2_O_2_ (100 μM), the CO release from 100 μM CORM-A1 was still diminished with an initial yield of 2% and a total yield of 4% (entry 2, Table 8). At higher concentrations of CORM-A1 (1 mM) in the presence of H_2_O_2_ (100 μM), the initial CO release yield was 7%, which is slightly higher than that of the reaction in water (entry 1, Table 8). Since H_2_O_2_ is commonly used to induce oxidative stress in biological studies and is produced intracellularly under stress, these results also suggest difficulties in interpreting the results when CORM-A1 is used as a CO surrogate in biological studies. The ability for CORM-A1 to act as a reducing agent may introduce new mechanisms that could change the CO release rate and overall CO yield. Although, it is well-established in textbook hydroboration reactions that H_2_O_2_ oxidizes the –BH_2_ moiety formed following reduction of an alkene by borane, further mechanistic studies are necessary to determine the different chemical interactions involving CORM-A1.

**Table 8:** Effect of H_2_O_2_ on CO production from CORM-A1 in 100 mM PBS (*n* = 3)

Entry	[CORM-A1]	Added reagent	Initial CO yield% (15 min)	Total CO yield% (20+ h)
1	1 mM	100 μM H_2_O_2_	7 ± 2.4	44 ± 9.9
2	100 μM	100 μM H_2_O_2_	2 ± 0.4	4 ± 1.2
3	100 μM	300 μM H_2_O_2_	0.8 ± 0.3	2 ± 1.2

#### Biological media

The preceding results suggest that the CO release from CORM-A1 is dependent on substrates and reagents present in solutions. Normal variations in intracellular concentrations of components such as NAD^+^, NADP^+^, metal ions, and peroxides may lead to substantial fluctuations of CO release rate and yield. As previously discussed, there are many emerging reports on the CO-independent effects of other CORMs in chemical and biological studies.^30,31,33,37,38,45^ Finally, there was one last, incredibly important aspect to probe: CO production from CORM-A1 in biological media.

##### CO production from CORM-A1 is dependent on the biological media used

Naturally when examining the biology of CO, studies using CO surrogates are often conducted in biological media. The idiosyncratic nature of CO production from CORM-A1 in the studies in simple solvents, as discussed thus far, compelled a brief investigation into its CO production in biological media. In solutions of DMEM containing 10% FBS, 1 mM CORM-A1 released low yields of CO similar to that in PBS solutions (∼18% in the first 15 minutes and 44% within 24 hours) (entry 1, Table 9). To our surprise, when incubated in RPMI1640 culture medium (without FBS) solutions, 1 mM CORM-A1 had significantly diminished CO production, 2% in the first 15 minutes and 41% within 24 hours (entry 2, Table 9). The mechanistic underpinnings of why the starved media led decreased initial CO production compared to media containing FBS is difficult to probe without knowing the exact composition of FBS. Determination of all the mechanistic differences proves to be inconsequential, as these results further show that CORM-A1 has unreliable CO production that seems to follow intractable patterns. Such results present a convoluted picture for result interpretation. The new data described should further encourage researchers to consider various variables and uncertain chemical reactivities when using CORM-A1 as a CO donor for studying CO biology.

**Table 9:** CO production from CORM-A1 in different biological media (*n* = 3)

Entry	[CORM-A1]	Media	Initial CO yield% (15 min)	Total CO yield% (20+ h)
1	1 mM	DMEM, 10% FBS	18 ± 2.9	44 ± 3.7
2	1 mM	RPMI1640 culture medium (without FBS)	2 ± 0.4	41 ± 1.1

### Consideration on the viability of CORM-A1 as a CO surrogate

In this section, we summarily address these issues described in various section with the aim of synthesizing a cohesive set of characteristics of CORM-A1 for easy correlation with implications in its use as a “CO surrogate.” Table 10 summaries key findings for discussion. First, the newly found redox reactions of CORM-A1 with biologically important molecules such as NAD^+^ and NADP^+^ raise concerns of its biological effects way beyond CO alone, *i.e.*, significant CO-independent effects. This is especially true considering the reported involvement of NAD(P)^+^ in CO-related signaling events. In addition, CORM-A1 had already been reported to scavenge ROS, which are known mediators of CO-related signaling events. The complexity of the redox issues seems hard to untangle in interpretating the CO-dependent effects from CORM-A1. Further, there are many other redox-active species involved in cellular functions including reactive sulfur species (RSS), reactive nitrogen species (RNS), and reactive electrophiles such as aldehydes (*e.g.*, glucose, pyridoxal/vitamin B_6_, retinal, pyruvate, among many others), which could be subjected to borane-mediated redox reactions. All these factors combined seem to make the redox reaction issue almost intractably complex in separating the putative CO-mediated biological response of CORM-A1 from that of chemical reactions. Second, there does not seem to a viable negative control to cancel the effects of chemical reactivity from CORM-A1 in biological studies. The traditionally used iCORM-A1 is essentially boric acid/borate, an oxidized form of BH_3_, which is devoid of the redox activity of BH_3_ under normal physiological conditions.

**Table 10:** Summary of factors that alter CORM-A1 CO production

Aspect probed	Reagents used	Effect on CO production
Buffer/phosphate anions	Phosphate buffered saline, lithium phosphate buffer	Concentration-dependent acceleration
CO-independent redox activity	NAD^+^, NADP^+^, NADH, 1-methylnicotinamide	Acceleration
Oxidants	H_2_O_2_, DMSO	Inhibition
Oxidants in DMSO	DDQ, ODQ	Reversal of inhibition; indicating chemical reactivity
Cations (organic and inorganic)	*N*-Methyl pyridine, *N*-methyl DABCO, Na^+^ (in PBS and NaCl) and Li^+^ (in lithium phosphate buffer)	No effect
Biological media	DMEM (10% FBS)	Acceleration (when compared to PBS)
Biological Media	RMPI (starved)	Decrease of initial CO production

A third point touches upon the most critical issue, *i.e.*, whether CORM-A1 releases a sufficient amount of CO within a reasonable period of time reproducibly and reliably to qualify CORM-A1 as a CO donor for studying CO biology. At this point, we start with the issue of reproducibility and reliability and then come back to the points of “sufficient amount” and “within a reasonable period of time.” This is because a lack of reproducibility and reliability renders the issue of “sufficient amount” and “within a reasonable period of time” moot points in analyzing CO-dependent effects from CORM-A1.

On the issue of “reproducibility and reliability,” it is important to highlight the salient CO-release features already presented. It should be noted that for a volatile molecule such as CO, its release rate from a given donor is a critical factor to consider because of its rapid exchange with the environment and the known effects of release rate on its sustained concentration in solution.^55^ The wide variability reported here implicates each future biological experiment to carefully determine CO release yields and kinetic profiles under the specific experimental conditions used before analyzing CO-dependent effects and dose–response relationship. The convoluted interplay of effects of various anions, cations, redox reactions, and biological media on CO release defies a unified mechanism for explanation and seems to be idiosyncratic. This level of perturbation of CO release yields by various factors make it extremely difficult to decipher CO-dependent effects when CORM-A1 is used as a CO donor. Further, the level of complexity of the intracellular environment and *in vivo* is guaranteed to be greater than the experimental conditions that we used in solution, which makes an intractable situation impossible to deconvolute. With the intractable nature of CO release, it is clear that using CORM-A1 as a CO donor in studying CO biology will be faced with many CO-independent problems. At this time, it almost seems inconsequential whether we know the mechanism(s) of actions of all the perturbation factors because these factors all lead to intractable problems.

In addition to the issue of overall suitability for CORM-A1 to be used as a CO donor for studying its biology, there is another important experimental question to address. Earlier reports state CORM-A1 being stable in water. There needs to be an important conversation on the definition of stability and what “stable” means in the case of CORM-A1. This is especially important if a stock solution of CORM-A1 in water is used in experiments. In our opinion, the adjective “stable” carries little meaning if not defined with numbers within the context of specific conditions and/or factors. The following discussions summarize the data presented in this work and show the idiosyncratic nature of CO production from CORM-A1 and the lack of stability for CORM-A1 stock solution to be used after hours of storage. The stability data presented in the Criterion III, Assessment 1 section clearly indicate that such a broad-stroke statement of “stable” is incorrect. First, in the case of unbuffered water solutions, where CORM-A1 has been reported to be stable previously, it still releases up to 33% within a 24 hour period. This inconsistency could alter the true concentration of CO that is delivered from a degrading stock solution. Second, the addition of certain reagents such as NAD(P)^+^ reverses CORM-A1's stated stability in unbuffered water. Third, the data suggest that CORM-A1 releases CO in a concentration-dependent manner with regard to phosphate anion, a concept yet to be discussed with the stability of CORM-A1. Finally, CO production from CORM-A1 is easily altered, whether accelerated or diminished, in the presence of simple reagents commonly encountered in bioassays such as peroxide, inorganic cations, and anions. Furthermore, one can only assume that the complex differences that exist in biological studies, in comparison to the simplicity of *in vitro* chemical reactivity studies, could only introduce more uncertainty in the use of CORM-A1 as an efficient CO donor.

Overall, the chemical reactivity and idiosyncratic nature of CO production from CORM-A1 in the presence of different substrates makes CORM-A1's role as a CO “surrogate” uncertain to say the least, as it fails to meet three basic criteria for a viable CO surrogate. There are already multiple reports on the CO-independent effects of other CORMs; therefore these results only further accentuate the complexities of chemical reactivity when using these chemically reactive CORMs with inadequate CO release under normal physiological conditions.^30,31,33,37,38,45^ With the understanding of the complexities and differences between biological and chemical studies, we urge the need to consider these chemical aspects when evaluating controls in studies using CORM-A1. Finally, to truly understand the mechanism on how various factors affect CO release from CORM-A1, much more extensive work is needed. However, if CORM-A1 is not a suitable CO donor for biological applications, the emphasis is to avoid its usage, not on additional resources being expended on studying the in-depth mechanism.

An additional point related to the idiosyncratic nature of CO release from CORM-A1 is its unfortunate use as the recommended standard in calibrating a certain commercially available CO electrode. In our hand, the electrode was unable to produce consistent and reproducible data after calibrating using CO gas instead of CORM-A1. We urge caution in quantitative studies when using any electrodes that recommend CORM-A1 as the standard for calibration.

## Conclusions

The results presented clearly demonstrated three key points. First, CORM-A1 does not reproducibly and reliable release an adequate amount of CO. Therefore, CORM-A1 fails the most basic test for it to be qualified as a CO surrogate in studying CO biology. Second, CORM-A1 has extensive chemical reactivity, leading to intractable chemical reaction problems, which is bound to result in CO-independent effects and potentially serious side effects. Third, there is no adequate control available in using CORM-A1 as a CO surrogate. All these new findings do not affect the reliability of the biological results from CORM-A1 in the literature, but they do affect interpretation of the results in the context of CO-related activity.

In view of the different issues discovered with regard to the four most widely used CORMs: CORM-2, CORM-3, CORM-401, and CORM-A1, we would like to urge consideration of the following issues in future work in developing new CO donors for studying CO biology. First, the CO donors need to be devoid of significant chemical reactivity, especially if such reactivity is non-selective and hard to control (*e.g.*, BH_3_ and Ru complexes). This is not a high bar, but very important. Second, CO release kinetics needs to be carefully defined under normal physiological conditions. Without a good understanding of the kinetics, CO concentration is hard to define at a given time even if the concentration of the donor molecule is known. Third, there needs to have at least one adequate control for the donor molecule. Another point of consideration is the release half-life of a donor molecule. Given the biological half-life of CO is 20 min in mice^56^ and a few hours in humans,^21,57^ a donor molecule with a release half-life much longer than CO's biological half-life is unlikely to lead to the delivery of a meaningful amount of CO.

We hope the work described in this manuscript will contribute significantly not only to improved understanding of the various properties of the four most commonly used CO donors (CORM-2, CORM-3, CORM-401, and CORM-A1), but also to future designs of new CO donors. We also hope our work will stimulate more research.

## Data availability

Supporting data for this article have been uploaded as part of the ESI material.†

## Author contributions

Nicola Bauer: Conceptualization, Methodology, Investigation, Validation, Writing- original draft preparation. Xiaoxiao Yang: Conceptualization, Methodology, Investigation, Writing-review and editing. Zhengnan Yuan: Conceptualization, Writing-review and editing. Binghe Wang: Conceptualization, Supervision, Writing-review and editing.

## Conflicts of interest

There are no conflicts to declare.

## Supplementary Material

SC-014-D3SC00411B-s001
