# Supplementary material for: Reassessing CORM-A1: redox chemistry and idiosyncratic CO-releasing characteristics of the widely used carbon monoxide donor
Source: Chem Sci. 2023 Feb 24;14(12):3215–28. doi: 10.1039/d3sc00411b (PMC10033827; doi:10.1039/d3sc00411b)
Supplement: SC-014-D3SC00411B-s001 [file SC-014-D3SC00411B-s001.pdf]

Electronic Supplementary Material (ESI) for Chemical Science.
This journal is © The Royal Society of Chemistry 2023

Supplementary Information

Reassessing CORM-A1: redox chemistry and idiosyncratic CO-releasing characteristics of the widely used carbon monoxide donor

Nicola Bauer, Xiaoxiao Yang, Zhengnan Yuan, and Binghe Wang*

Department of Chemistry and Center for Diagnostics and Therapeutics, Georgia State University, Atlanta, Georgia 30303, USA

Table of Contents

**<u>Experimental</u>**	Pages 2-6
a. General Information	2
b. UV-Vis Analysis	2-3
i. NAD(P)H standard curve (Fig S1)	3
c. LC-MS/MS Analysis	3-4
d. Quantatative CO Analysis	4
i. CO standard curves (Fig S2a-b)	4
ii. Equations used to calculate CO release	6
e. Reactions of 1-methylnicotinamide and CORM-A1 or $NaBH_4$ (Fig S3)	6
**LC-MS Chromatographs (Fig S4-S18)**	Pages 7-22
**Figure S4a-b**. XIC of 1mM NAD^+ standard in water detecting for NAD^+ (a) and NADH (b)	8
**Figure S5a-b**. XIC of 2mM NAD^+ standard in water detecting for NAD^+(a) and NADH (b)	9
**Figure S6a-b**. XIC of 2mM NAD^+ standard in THF detecting for NAD^+(a) and NADH (b)	10
**Figure S7a-b**. XIC of 1:1 CORM-A1:NAD^+ at 15min detecting for NAD^+(a) and NADH (b) (Fig 8a-b: duplication)	11-12
**Figure S9a-b**. XIC of 1:1 CORM-A1:NAD^+ at 30min detecting for NAD^+(a) and NADH (b) (Fig 10a-b: duplication)	13-14
**Figure S11a-b**. XIC of 10:1 CORM-A1:NAD^+ at 15min detecting for NAD^+(a) and NADH (b) (Fig 12a-b: duplication)	15-16
**Figure S13a-b**. XIC of 10:1 Boric Acid:NAD^+ at 15min detecting for NAD^+(a) and NADH (b) (Fig 14a-b: duplication)	17-18
**Figure S15a-b**. XIC of 10:1 BH_3-THF:NAD^+ at 15min detecting for NAD^+(a) and NADH (b) (Fig 16a-b: duplication)	19-20
**Figure S17a-b**. XIC of 2 mL CO gas and 1 mM NAD+ at 15min detecting for NAD^+(a) and NADH (b) (Fig 18a-b: duplication)	21-22
**Figures S19. ^{11}B NMR of CORM-A1 and NAD^+ (3:1, 1:1) in D_2O**	23
References	23

Experimental

General Information. All reagents and solvents were of reagent grade from commercial suppliers (Sigma-Aldrich, etc.). Absorption spectra were measured and observed at 340 nm (NADH) and 260 nm (NAD^+) on Varian Cary 100 Bio UV−visible spectrophotometer (UV-Vis). Liquid chromatography−mass spectrometry (LC−MS/MS) data were collected on an API 3200 LC/MS/MS system, a triple quadruple mass spectrometer coupled with an Agilent 1200 series HPLC and UV-DAD. Gas chromatography studies were performed on an Agilent 7820A system equipped with flame ionization detector (FID) coupled with a methanizer (CH4izer, Restek, USA). Column: Packed; 80/100 5A molecular sieve solid support, L × I.D. 2m × 0.53 mm (Restek). Carrier gas: Helium (Airgas). Gas tanks (Helium, H_2, CO, and Air) were purchased from Airgas.. CORM-A1, nicotinamide adenine dinucleotide (NAD^+), reduced nicotinamide adenine dinucleotide (NADH), nicotinamide adenine dinucleotide phosphate ($NADP^+$), reduced nicotinamide adenine dinucleotide phosphate (NADPH), 1-methylnicotinamide chloride, $NaBH_4$ and 1M BH_3-THF were purchased from Sigma-Aldrich and were used without purification. Boric acid, phosphate buffer saline (1×, 10×, 20×), DMEM (Corning, New York, USA), and RPMI 1640 (Corning, New York, USA) culture medium were purchased from Fischer Scientific. Sodium chloride (NaCl) and ODQ (1H-[1,2,4]oxadiazolo[4,3-a]-quinoxalin-1-one) were purchased from VWR. Hydrogen peroxide (35%, H_2O_2) was purchased from Oakwood Chemicals. *N*-Methylpyridine and methylated DABCO were synthesized using literature procedures.[1, 2] Lithium phosphate buffer was made using phosphoric acid and lithium hydroxide. Pure CO gas was purchased from Airgas Company.

UV-Vis Analysis. Absorption spectra were measured and observed at 340 nm (NADH) and 260 nm (NAD+) on Varian Cary 100 Bio UV−visible spectrophotometer (UV-Vis) using a 1-mL cuvette. Standard curves for NADH and NADPH were established to calculate the approximate concentration of NADH produced in each reaction (Fig 2 (and Fig. S2). The reactions were either incubated at higher concentrations, specified for each experiment as described in the main text, and then diluted with PBS to 100-200 μM to maintain an absorbance under 1.0 au or conducted at 100-200 μM. For reactions conducted at higher concentrations (e.g., 2 mM CORM-A1: 2 mM NAD^+), CORM-A1 was weighed and the solid was placed into a glass vial. The necessary volume to obtain the reported concentration of CORM-A1 was added through a solution of NAD^+ or $NADP^+$. The glass vial was then incubated at 37 °C using a water bath and diluted to 1-mL at each designated time point. For reactions conducted at 100-200 μM, CORM-A1 was weighed and the solid was directly added to solution in cuvette (heated to 37 °C) and reaction was monitored. *Hence, a stock solution of CORM-A1 was not used in any UV-Vis experiments, since the CORM-A1 solid was directly added to all solutions.*

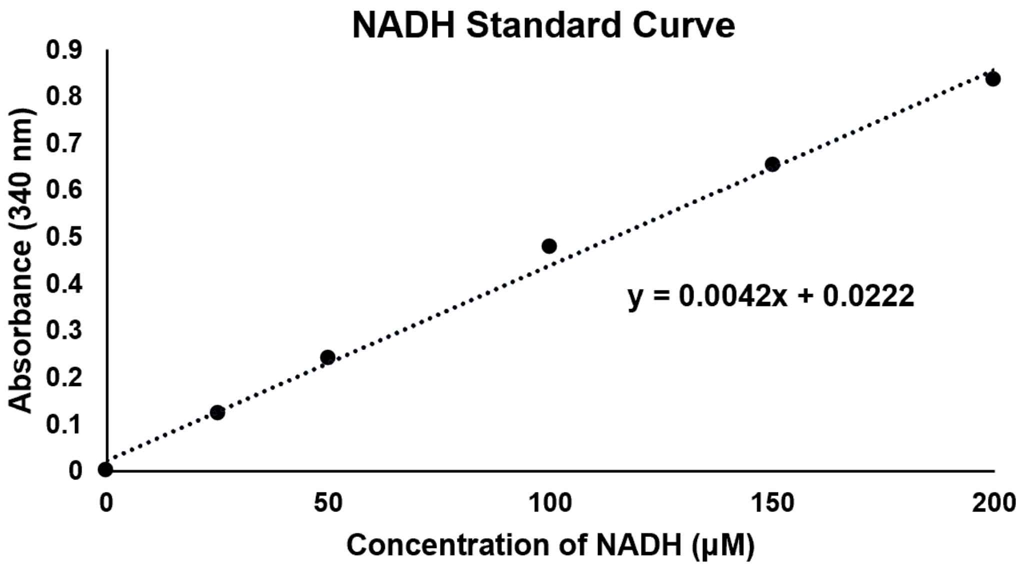

Figure S1A. *NADH Standard Curve in PBS*

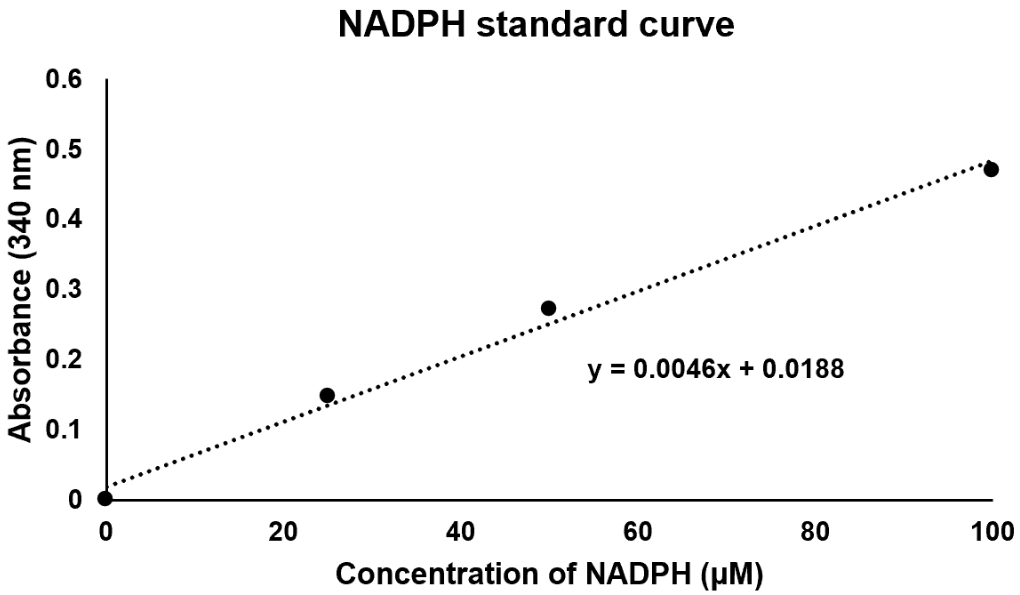

Figure S1B. *NADPH Standard Curve in PBS*

LC-MS/MS Analysis. LC-MS/MS Data was obtained using an API 3200 triple quadruple mass spectrometer coupled with an Agilent 1200 series HPLC and UV-DAD spectrometer. Methods used a reversed-phase analytical column (Kromasil 100-3.5-C18) and electrospray ionization source in a negative mode with positive polarity. Selected ion mode was used for the detection of ions at m/z 662 and 540 for NAD^+ and m/z 664, 408, and 397 for

NADH. A 10-µL sample solution was delivered through automatic injection to the HPLC. The mobile phase was ACN and 0.1% formic acid (in water) used for gradient elution from 0-95-0% over 15 min at 500 µL/min flow rate. The CDL temperature was 200 °C and the heat block temperature was 250 °C. Nebulizing gas flow was 4.5 L/min. NAD^+: DP=50; EP=-10; CE=-26; CXP=-5; IS=4500; CAP=12; TEM=250; NADH: DP=-50; EP=-10; CE=-50; CXP=-5; IS=-4500.

General Protocol. Using a microbalance, NAD^+, NADH, CORM-A1, boric acid, and $NaBH_4$ were weighed and dissolved to create stock solutions in HPLC grade water (filtered and degassed via Barnstead NanoPure, pH: 5.5). Each were diluted using degassed and filtered water to desired concentrations. Solutions of BH_3-THF were measured via pipette and prepared in anhydrous THF solvent. For reactions, equal volumes of NAD^+ (either 4 mM or 2 mM) and boron-based compounds (either 4 mM or 20 mM) were added to HPLC-specific vials and incubated at 37 °C for either 15 or 30 minutes. For 1:1 molar ratio, 4 mM of each were added in equal volume to give a final concentration of 2 mM. For 10:1 molar ratio, 2 mM of NAD^+ and 20 mM of a boron-based compound were added in equal volume to give final concentrations of 1 mM and 10 mM, respectively. The ratio of NADH and NAD^+ is calculated by using AUC provided by the LC-MS, which included negligible background noise or impurity in the multi-reaction monitoring (MRM) spectra detecting for NADH, making up <2% of NADH achievable in reactions (Fig. S3b, S4b, S5b).

^{11}B NMR analysis. ^{11}B NMR spectra were recorded on Bruker-400 spectrometers (128 MHz for ^{11}B NMR). 100 mM CORM-A1 in D_2O and reaction of CORM-A1 and NAD^+ at 3:1 (100mM:33.3mM) and 1:1 (100mM:100mM) ratios are seen at the end of the SI in figure S19.

Quantitative CO Analysis. An Agilent 7820A GC System equipped with a methanizer-FID (CH4izer, Restek, USA) was used to quantify CO release yield of CORM-A1. Because of CORM-A1's originally reported fast CO release, along with our data describing degrading stock solutions of CORM-A1, it is important to emphasize *that stock solutions of CORM-A1 were not be used in any GC experiments*. Instead, CORM-A1 was weighed using a microbalance, and the solid was then sealed in a gas tight headspace glass vial. The designated solution, including solvent and added reagent (e.g. NAD^+, $NADP^+$, etc.), was then added to sealed vial via a syringe. Using a gas tight syringe, 100 µL of the headspace of 6-mL (actually 8.8-mL) Supelco headspace vials were sampled and transferred to the injector port maintained at 150 °C. Helium was used as the carrier gas with a flow rate of 30 mL/min. Gaseous components of the headspace were separated by passing through a packed column with 80/100 5A molecular sieve solid support, L × I.D. 2m × 0.53 mm (Restek). The column was heated at 100 °C for 5 min then 250 °C for 10 min while the detector was held at 300 °C. To calculate the CO release yield from CORM-A1, a standard curve was established using pre-made CO gas of 10, 20, 50, 100, and 10,000 ppm (Figure S2a). Over the course of the study, the catalyst in the methanizer required replacement and another standard curve was generated under the same condition (Figure S2b) after catalyst replacement to ensure the system suitability. The AUC observed was used in a

linear equation to solve for concentration of CO released from CORM-A1. CO yield was calculated using Equation S1.

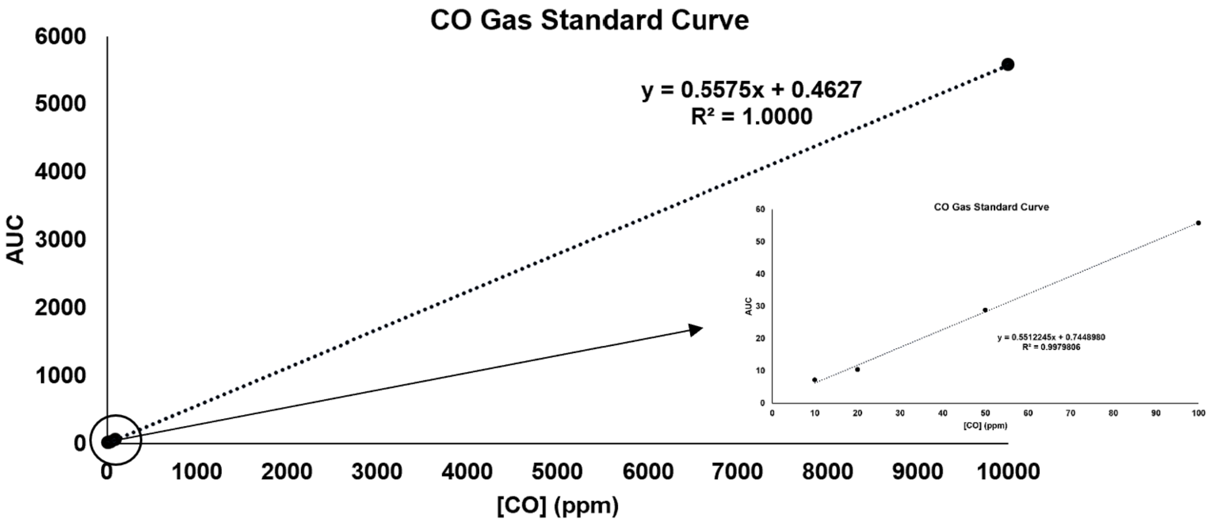

Figure S2a. *CO standard curve using 100 µL injections*

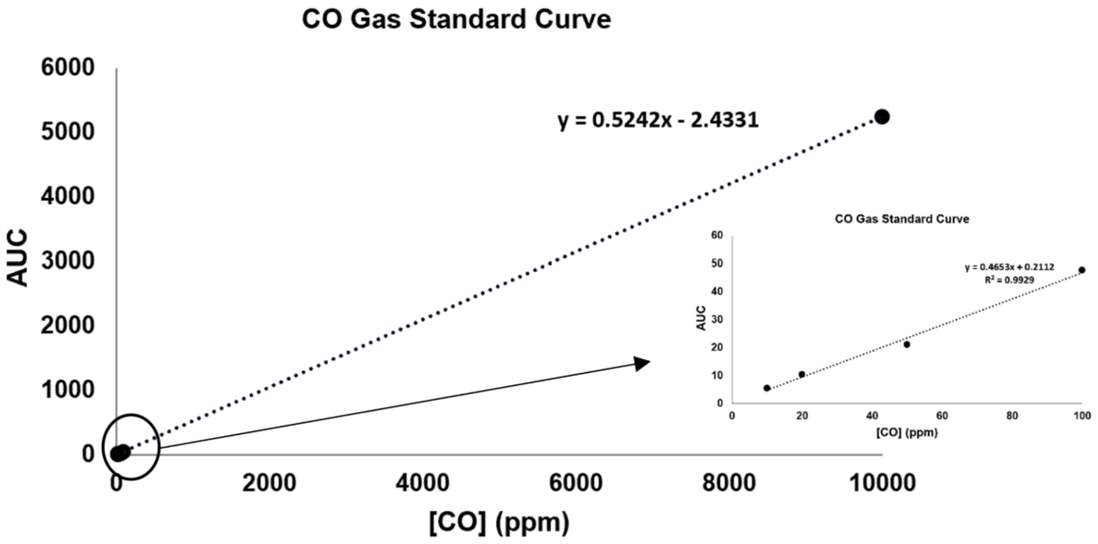

Figure S2b. *CO standard curve (after catalyst replacement) using 100 µL injections*

$$CO\ (PPM) = \frac{AUC}{slope}\ (simplified\ by\ using\ CO\ (PPM) = AUC\ x\ 2)$$

$$CO\ (mol) = \frac{(CO\ (PPM)\ x\ 10^{-6})\ x\ L\ headspace}{22.4}$$

$$CO\ Yield\ \% = \left(\frac{mol\ of\ CO\ experimental}{mol\ of\ CO\ theoretical}\right) x\ 100$$

Equation S1. *Equations used to calculate CO yield from CORM-A1. L headspace is calculated by subtracting the amount of solvent added from the total volume of the vial (8.8-mL)*

Table S1. CO Production from 1 mM CORM-A1 in 100mM PBS at room temperature

Entry	[CORM-A1]	Solvent	Added Reagent:	Initial CO Yield % (15 min)	Total CO Yield % (20+ h)
**S1**	1 mM	10× PBS (at RT)	---	13 ± 3.2	54 ± 4.0

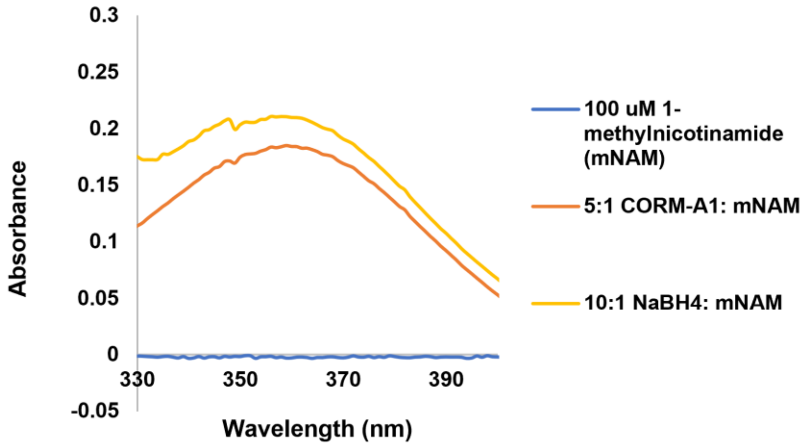

Figure S3. *Reactions of 1-methylnicotinamide (1 mM) and CORM-A1 (5 mM) or $NaBH_4$ (10 mM) in PBS, monitored by UV-Vis after 10-fold dilution of the reaction solution. PBS sample shown to represent no change in absorbance from the blank and mNAM control.*

LC-MS Chromatographs

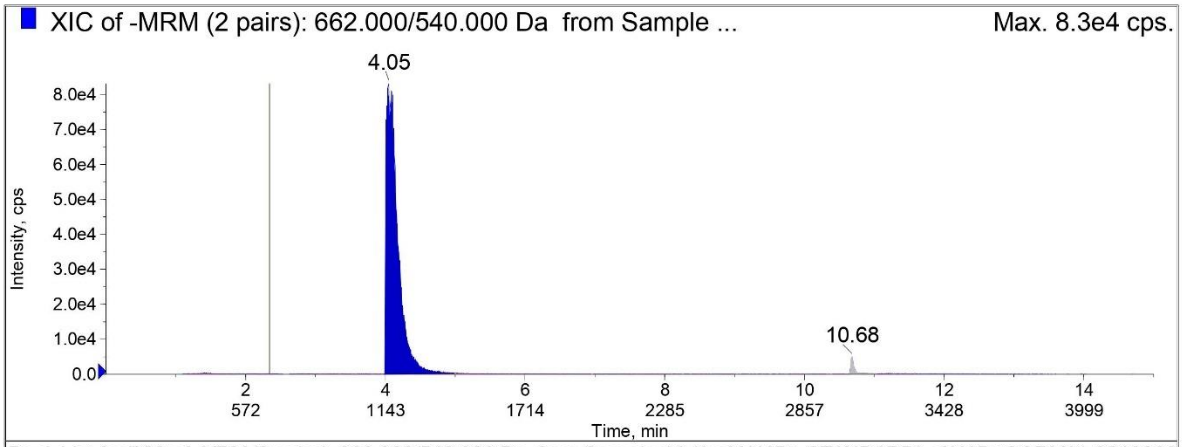

Peak List for "XIC of -MRM (2 pairs): 662.000/540.000 Da from Sample 14 (1mM NAD+ STANDARD) of NAD+CORMA1-0210202

	Time (min)	Area (counts)	%Area	Height (cps)	%Height	Width (min)	Baseline Type
1	4.0513	9.4737e5	98.1232	8.3248e4	94.3112	0.9980	Base to Base
2	10.6827	1.8120e4	1.8768	5021.5227	5.6888	0.3782	Base to Base

Figure S4a. *XIC of 1mM NAD$^+$ standard in water detecting for NAD$^+$*

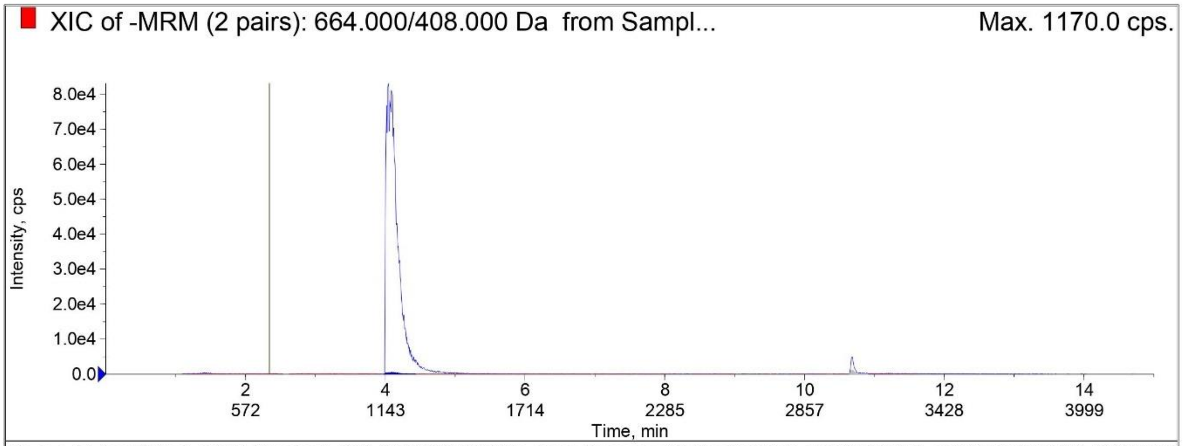

Peak List for "XIC of -MRM (2 pairs): 664.000/408.000 Da from Sample 14 (1mM NAD+ STANDARD) of NAD+CORMA1-0210202

	Time (min)	Area (counts)	%Area	Height (cps)	%Height	Width (min)	Baseline Type
1	4.0998	5986.4800	56.3496	578.4500	33.4664	0.4587	Base to Base
2	10.6773	4637.3350	43.6504	1150.0000	66.5336	0.2697	Base to Base

Figure S4b. *XIC of 1mM NAD$^+$ standard in water detecting for NADH*

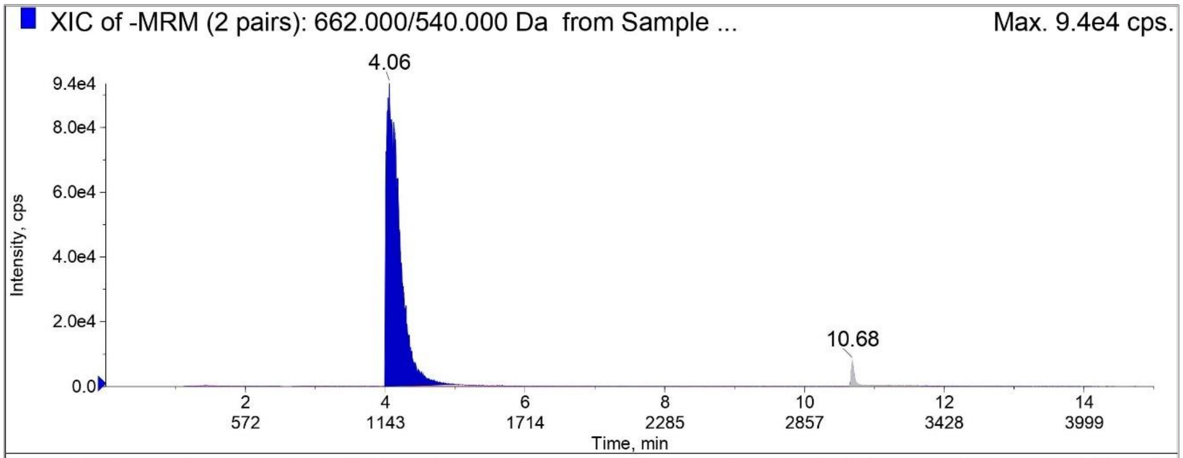

Peak List for "XIC of -MRM (2 pairs): 662.000/540.000 Da from Sample 11 (2mM NAD+ STANDARD) of NAD+CORMA1-0210202

	Time (min)	Area (counts)	%Area	Height (cps)	%Height	Width (min)	Baseline Type
1	4.0594	1.2171e6	97.2681	9.3717e4	92.1386	1.0050	Base to Base
2	10.6844	3.4184e4	2.7319	7996.0830	7.8614	1.0086	Base to Base

Figure S5a. *XIC of 2mM NAD$^+$ standard in water detecting for NAD$^+$*

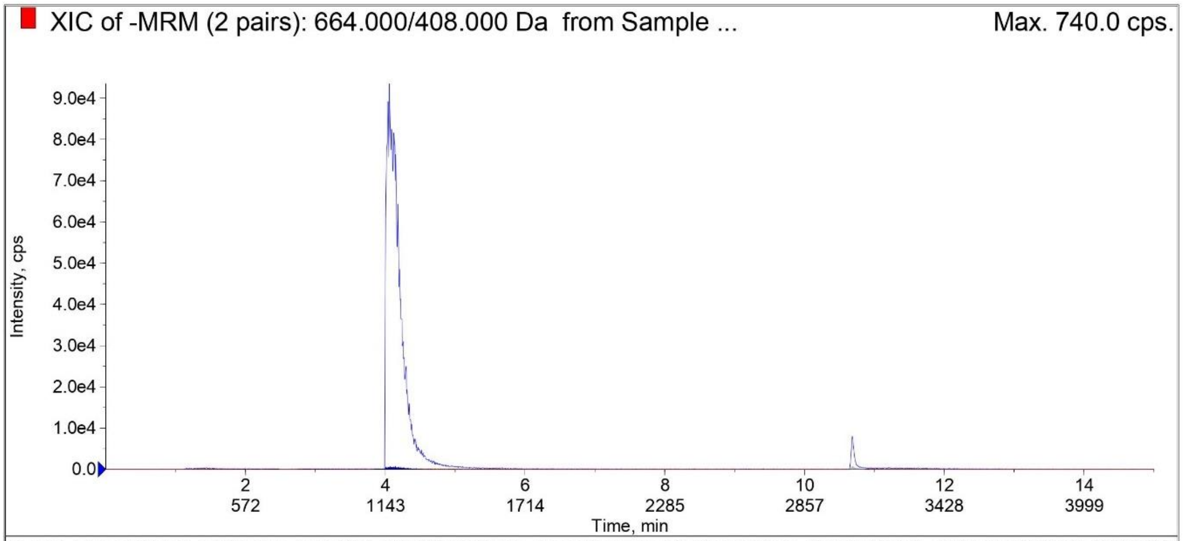

Peak List for "XIC of -MRM (2 pairs): 664.000/408.000 Da from Sample 11 (2mM NAD+ STANDARD) of NAD+CORMA1-0210202

	Time (min)	Area (counts)	%Area	Height (cps)	%Height	Width (min)	Baseline Type
1	4.0740	7014.5100	71.6499	627.4315	46.0114	0.6093	Base to Base
2	10.6933	2775.4650	28.3501	736.2134	53.9886	0.2066	Base to Base

Figure S5b. *XIC of 2mM NAD$^+$ standard in water detecting for NADH*

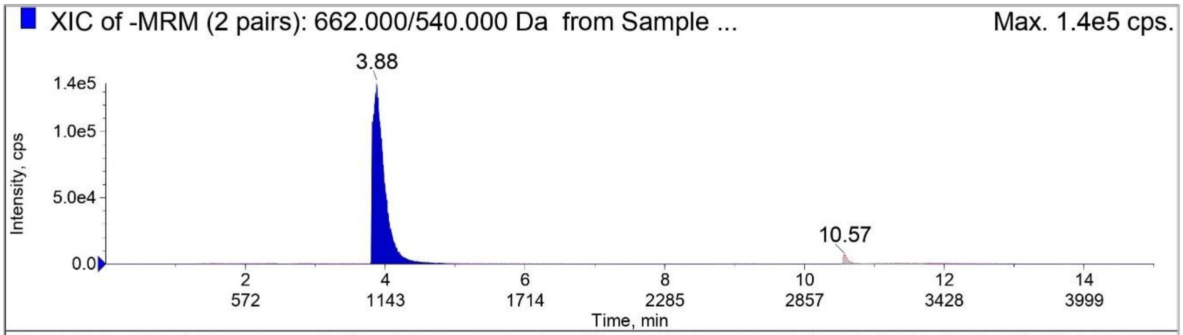

Peak List for "XIC of -MRM (2 pairs): 662.000/540.000 Da from Sample 27 (NAD+1mM-STANDARD) of NAD+CORMA1-0210202[illegible]

	Time (min)	Area (counts)	%Area	Height (cps)	%Height	Width (min)	Baseline Type
1	3.8777	1.5366e6	97.5635	1.3734e5	95.9487	1.0926	Base to Base
2	10.5736	3.8374e4	2.4365	5798.7949	4.0513	1.1871	Base to Base

Figure S6a. *XIC of 1mM NAD$^+$ standard in THF detecting for NAD$^+$*

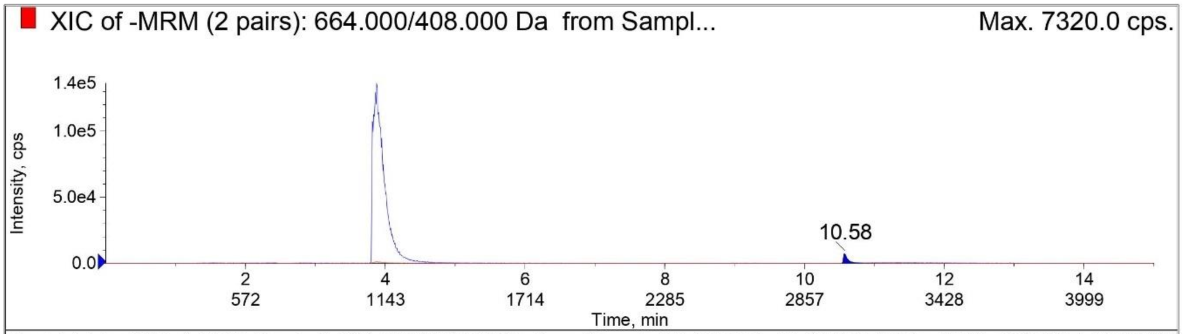

Peak List for "XIC of -MRM (2 pairs): 664.000/408.000 Da from Sample 27 (NAD+1mM-STANDARD) of NAD+CORMA1-0210202[illegible]

	Time (min)	Area (counts)	%Area	Height (cps)	%Height	Width (min)	Baseline Type
1	10.5800	3.2507e4	100.0000	7317.2772	100.0000	0.3012	Valley

Figure S6b. *XIC of 1mM NAD$^+$ standard in THF detecting for NADH*

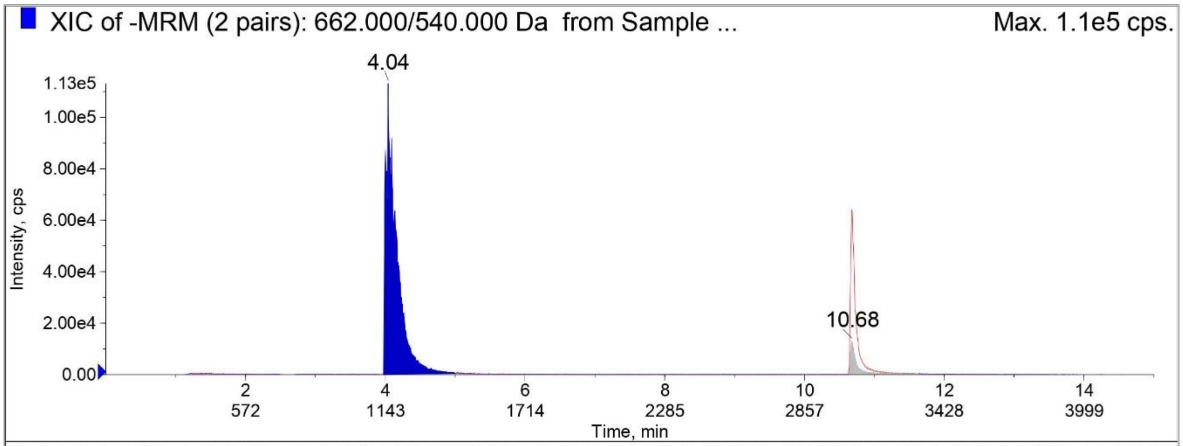

Peak List for "XIC of -MRM (2 pairs): 662.000/540.000 Da from Sample 9 (1:1 NAD+ CORMA1 15 MIN) of NAD+CORMA1-021020

	Time (min)	Area (counts)	%Area	Height (cps)	%Height	Width (min)	Baseline Type
1	4.0420	1.1623e6	93.4706	1.1495e5	89.8992	1.0295	Base to Base
2	10.6815	8.1193e4	6.5294	1.2916e4	10.1008	1.0155	Base to Base

***Figure S7a**. XIC of 1:1 CORM-A1:NAD^{+} at 15min detecting for NAD^{+}*

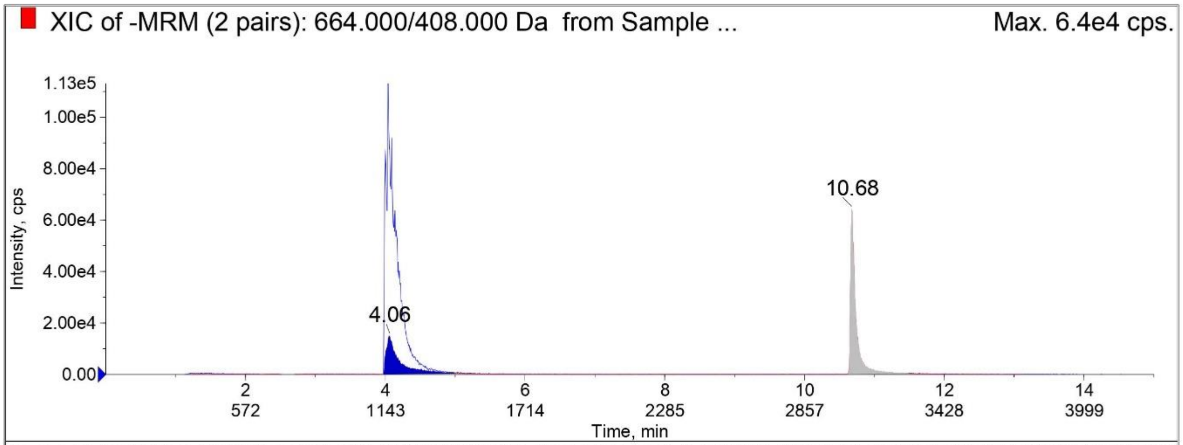

Peak List for "XIC of -MRM (2 pairs): 664.000/408.000 Da from Sample 9 (1:1 NAD+ CORMA1 15 MIN) of NAD+CORMA1-021020

	Time (min)	Area (counts)	%Area	Height (cps)	%Height	Width (min)	Baseline Type
1	4.0620	1.7938e5	34.9038	1.4765e4	18.7253	1.0050	Base to Base
2	10.6775	3.3454e5	65.0962	6.4085e4	81.2747	0.8544	Valley

***Figure S7b**. XIC of 1:1 CORM-A1:NAD^{+} at 15min detecting for NADH*

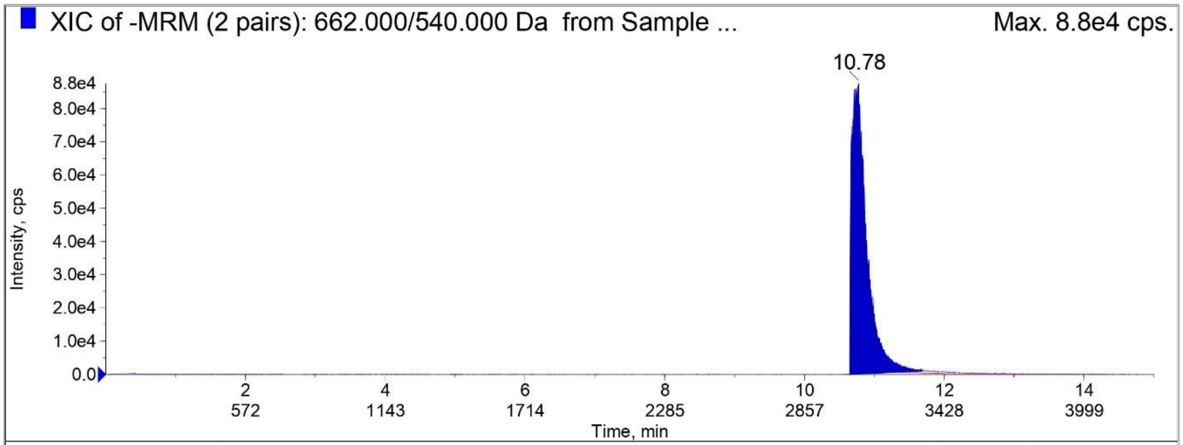

Peak List for "XIC of -MRM (2 pairs): 662.000/540.000 Da from Sample 12 (1:1 NAD+ CORMA1 15 MIN-2) of NAD+CORMA1-021[illegible]

	Time (min)	Area (counts)	% Area	Height (cps)	% Height	Width (min)	Baseline Type
1	10.7754	1.3032e6	100.0000	8.7468e4	100.0000	1.0506	Base to Base

Figure S8a. *XIC of 1:1 CORM:NAD+ (2nd) at 15min detecting for NAD*$^+$

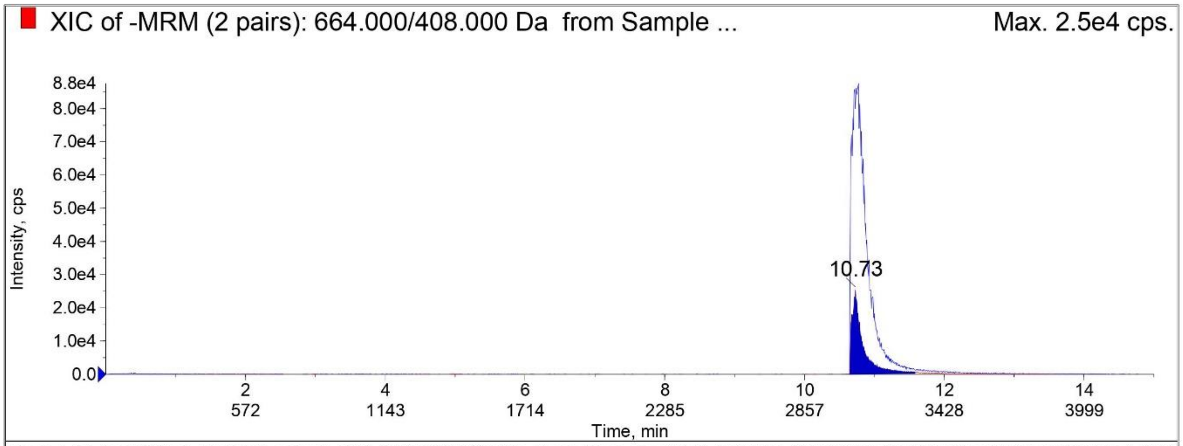

Peak List for "XIC of -MRM (2 pairs): 664.000/408.000 Da from Sample 12 (1:1 NAD+ CORMA1 15 MIN-2) of NAD+CORMA1-021[illegible]

	Time (min)	Area (counts)	% Area	Height (cps)	% Height	Width (min)	Baseline Type
1	10.7301	2.6017e5	100.0000	2.5325e4	100.0000	0.9420	Valley

Figure S8b. *XIC of 1:1 CORM:NAD*$^+$*(2*nd*) at 15min detecting for NADH*

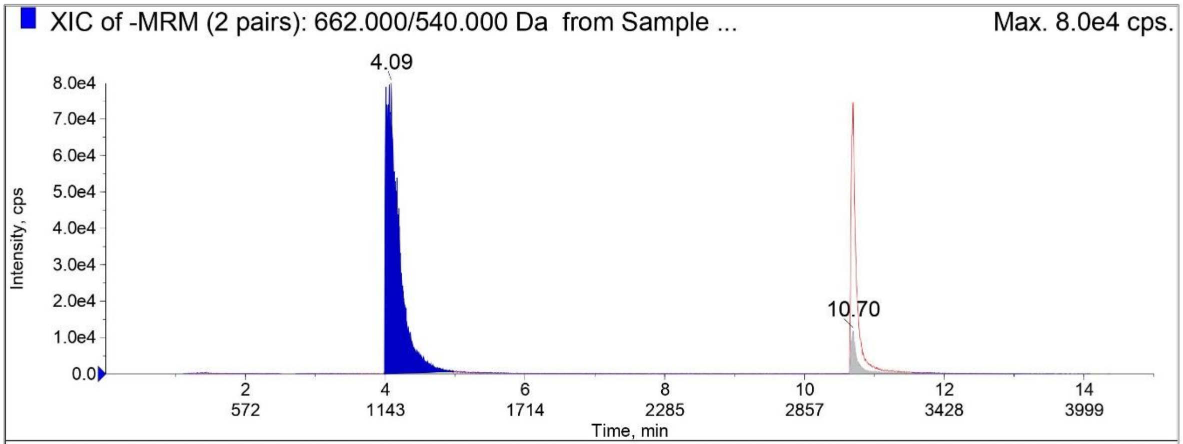

Peak List for "XIC of -MRM (2 pairs): 662.000/540.000 Da from Sample 10 (1:1 NAD+ CORMA1 30 MIN) of NAD+CORMA1-02102

	Time (min)	Area (counts)	%Area	Height (cps)	%Height	Width (min)	Baseline Type
1	4.0870	1.0353e6	93.2066	7.9809e4	87.0595	0.9981	Base to Base
2	10.6961	7.5461e4	6.7934	1.1863e4	12.9405	0.8895	Valley

***Figure S9a**. XIC of 1:1 CORM-A1:NAD^{+} at 30min detecting for NAD^{+}*

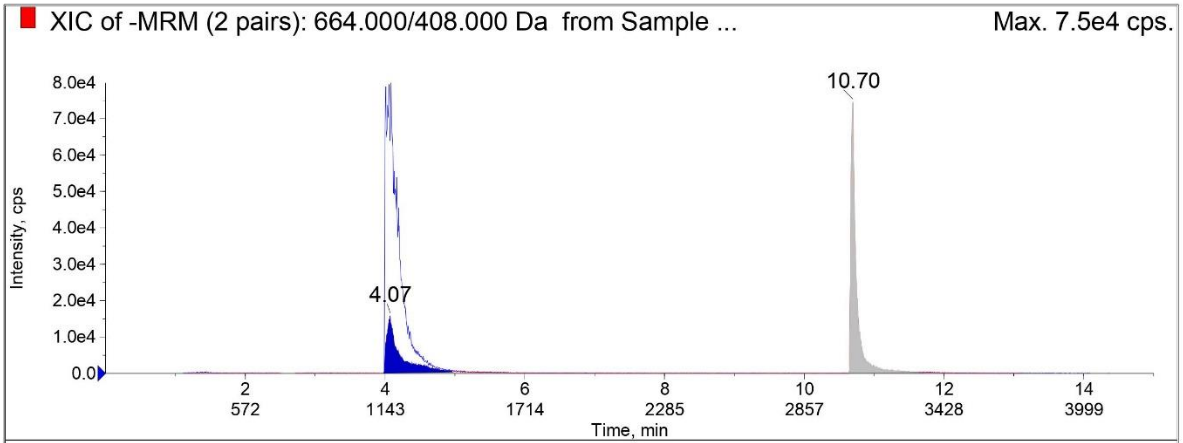

Peak List for "XIC of -MRM (2 pairs): 664.000/408.000 Da from Sample 10 (1:1 NAD+ CORMA1 30 MIN) of NAD+CORMA1-02102

	Time (min)	Area (counts)	%Area	Height (cps)	%Height	Width (min)	Baseline Type
1	4.0736	1.9142e5	32.3630	1.5835e4	17.4858	0.9840	Base to Base
2	10.6976	4.0007e5	67.6370	7.4726e4	82.5142	0.9980	Base to Base

***Figure S9b**. XIC of 1:1 CORM-A1:NAD^{+} at 30min detecting for NADH*

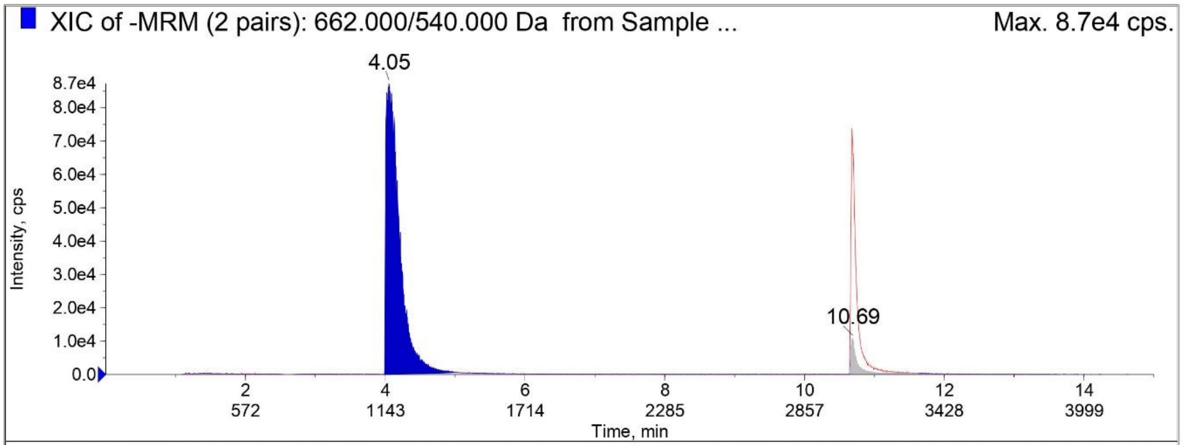

Peak List for "XIC of -MRM (2 pairs): 662.000/540.000 Da from Sample 13 (1:1 NAD+ CORMA1 30 MIN-2) of NAD+CORMA1-021[illegible]

	Time (min)	Area (counts)	%Area	Height (cps)	%Height	Width (min)	Baseline Type
1	4.0531	1.1600e6	94.2778	8.7966e4	89.0298	0.9980	Base to Base
2	10.6905	7.0406e4	5.7222	1.0839e4	10.9702	0.9840	Base to Base

Figure S10a. *XIC of 1:1 CORM-A1:NAD$^+$ (2nd) at 30min detecting for NAD$^+$*

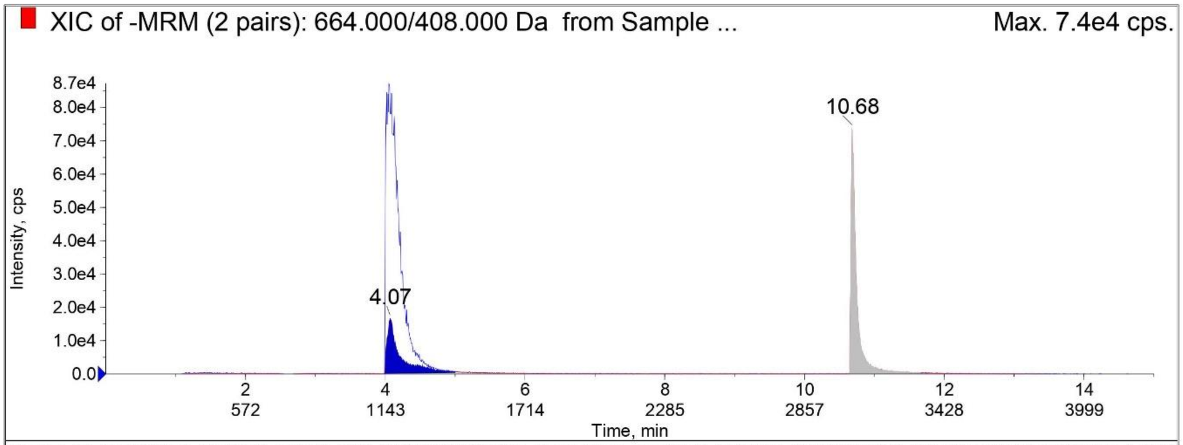

Peak List for "XIC of -MRM (2 pairs): 664.000/408.000 Da from Sample 13 (1:1 NAD+ CORMA1 30 MIN-2) of NAD+CORMA1-021[illegible]

	Time (min)	Area (counts)	%Area	Height (cps)	%Height	Width (min)	Baseline Type
1	4.0693	1.9914e5	33.3883	1.6502e4	18.1876	1.0085	Base to Base
2	10.6819	3.9730e5	66.6117	7.4230e4	81.8124	1.0226	Base to Base

Figure S10b. *XIC of 1:1 CORM-A1:NAD$^+$ (2nd) at 30min detecting for NADH*

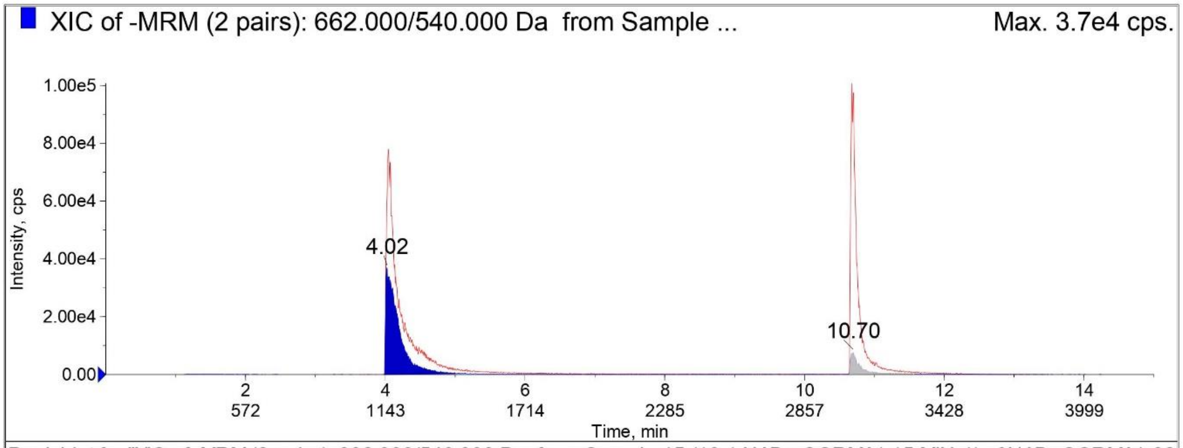

Peak List for "XIC of -MRM (2 pairs): 662.000/540.000 Da from Sample 15 (10:1 NAD+ CORMA1 15 MIN-1) of NAD+CORMA1-02

	Time (min)	Area (counts)	%Area	Height (cps)	%Height	Width (min)	Baseline Type
1	4.0246	4.5943e5	87.2826	3.6838e4	82.6924	1.0156	Base to Base
2	10.6957	6.6941e4	12.7174	7710.1651	17.3076	0.9245	Base to Base

Figure S11a. *XIC of 10:1 CORM-A1:NAD$^+$ at 15min detecting for NAD$^+$*

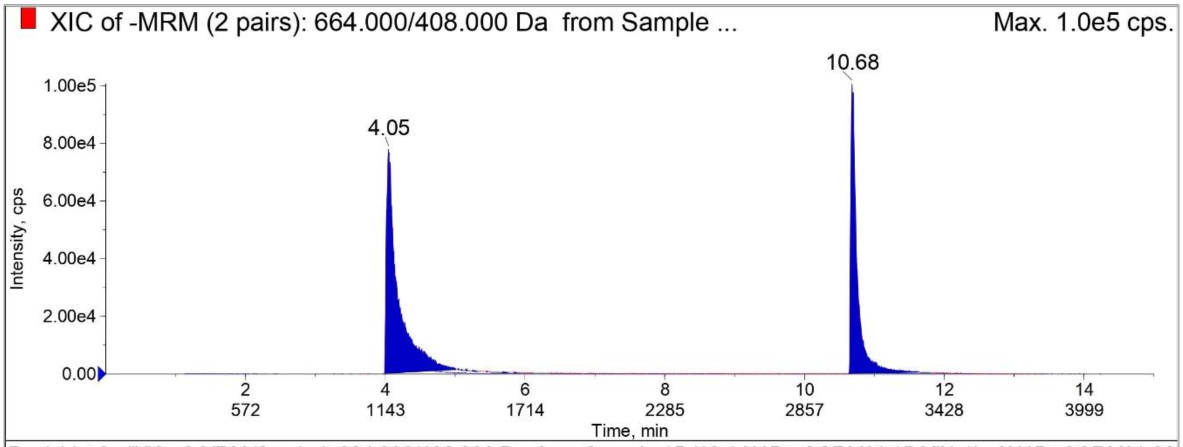

Peak List for "XIC of -MRM (2 pairs): 664.000/408.000 Da from Sample 15 (10:1 NAD+ CORMA1 15 MIN-1) of NAD+CORMA1-02

	Time (min)	Area (counts)	%Area	Height (cps)	%Height	Width (min)	Baseline Type
1	4.0479	8.8417e5	57.5843	7.7802e4	41.8090	1.0226	Base to Base
2	5.0346	206.0550	0.0134	551.1191	0.2962	0.0140	Base to Base
3	5.0436	350.7550	0.0228	377.9461	0.2031	0.0245	Base to Base
4	5.0690	133.5050	8.6949e-3	352.4688	0.1894	0.0140	Base to Base
5	5.0917	382.0800	0.0249	447.0157	0.2402	0.0245	Base to Base
6	5.1409	460.2850	0.0300	471.1149	0.2532	0.0385	Base to Base
7	5.1689	586.3650	0.0382	700.8130	0.3766	0.0280	Base to Base
8	5.2002	818.4250	0.0533	470.6461	0.2529	0.0560	Base to Base
9	5.2694	861.5500	0.0561	458.9457	0.2466	0.0735	Base to Base
10	5.3443	512.7300	0.0334	364.0541	0.1956	0.0455	Base to Base
11	5.4525	207.8600	0.0135	330.4243	0.1776	0.0280	Base to Base
12	5.5957	2584.4400	0.1683	492.9925	0.2649	0.1716	Base to Base

Figure S11b. *XIC of 10:1 CORM-A1:NAD$^+$ at 15min detecting for NADH*

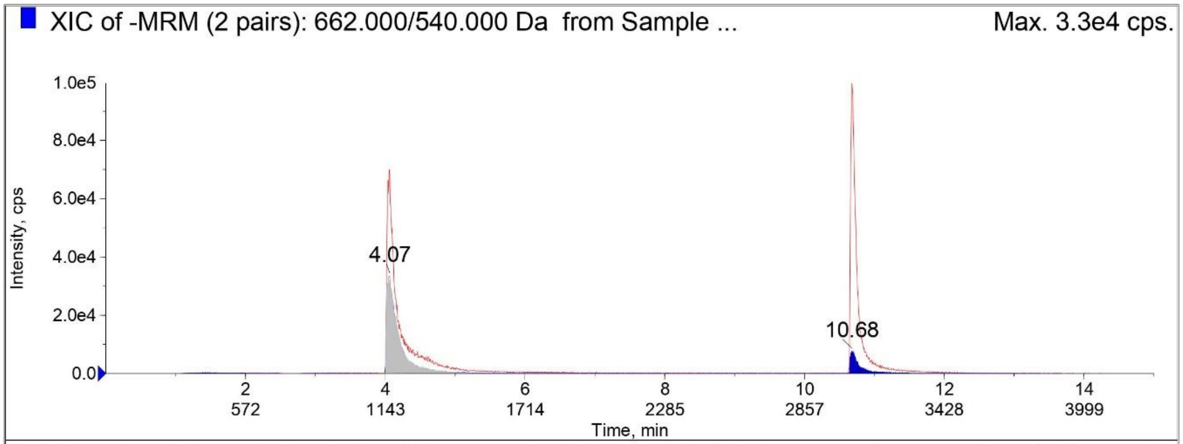

Peak List for "XIC of -MRM (2 pairs): 662.000/540.000 Da from Sample 16 (10:1 NAD+ CORMA1 15 MIN-2) of NAD+CORMA1-02

	Time (min)	Area (counts)	%Area	Height (cps)	%Height	Width (min)	Baseline Type
1	1.4213	5211.0500	1.1594	311.0227	0.7523	0.8649	Base to Base
2	4.0654	3.8053e5	84.6645	3.3398e4	80.7855	1.0436	Base to Base
3	10.6768	6.3715e4	14.1761	7632.5072	18.4622	0.8615	Base to Base

***Figure S12a**. XIC of 10:1 CORM-A1:NAD$^+$ (2nd) at 15min detecting for NAD$^+$*

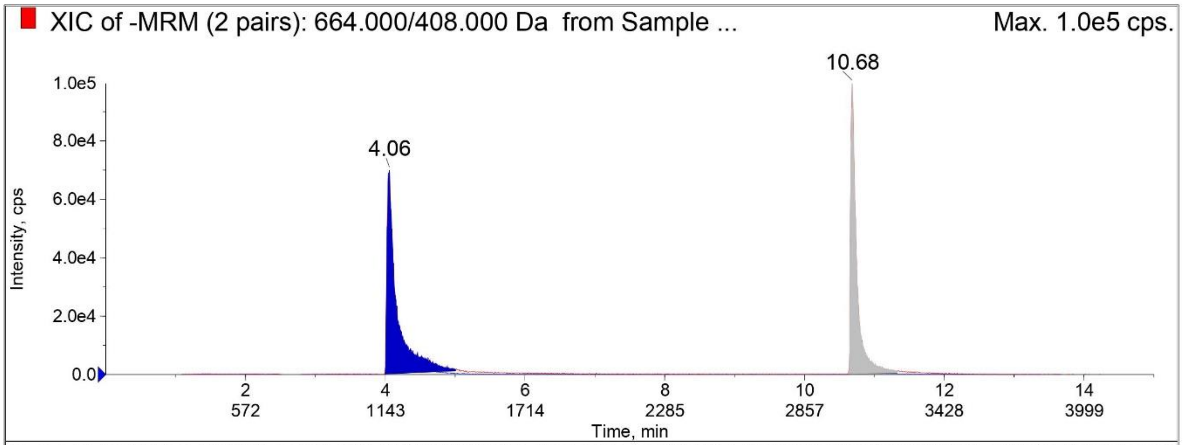

Peak List for "XIC of -MRM (2 pairs): 664.000/408.000 Da from Sample 16 (10:1 NAD+ CORMA1 15 MIN-2) of NAD+CORMA1-02

	Time (min)	Area (counts)	%Area	Height (cps)	%Height	Width (min)	Baseline Type
1	4.0591	6.8273e5	53.1233	6.9827e4	40.8456	1.0121	Base to Base
2	10.6825	6.0245e5	46.8767	1.0113e5	59.1544	0.6933	Valley

***Figure S12b**. XIC of 10:1 CORM-A1:NAD$^+$ (2nd) at 15min detecting for NADH*

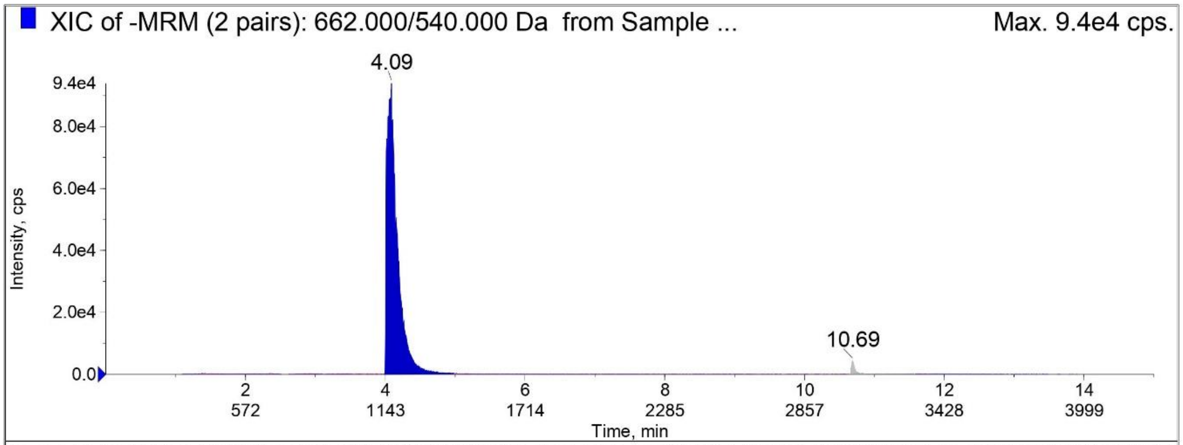

Peak List for "XIC of -MRM (2 pairs): 662.000/540.000 Da from Sample 17 (10:1 BORIC ACID 15 MIN -1) of NAD+CORMA1-0210

	Time (min)	Area (counts)	%Area	Height (cps)	%Height	Width (min)	Baseline Type
1	4.0896	9.7393e5	98.0057	9.4247e4	95.5964	0.9805	Base to Base
2	10.6884	1.9818e4	1.9943	4341.4087	4.4036	0.9245	Base to Base

***Figure S13a**. XIC of 10:1 Boric Acid:NAD^{+} at 15min detecting for NAD^{+}*

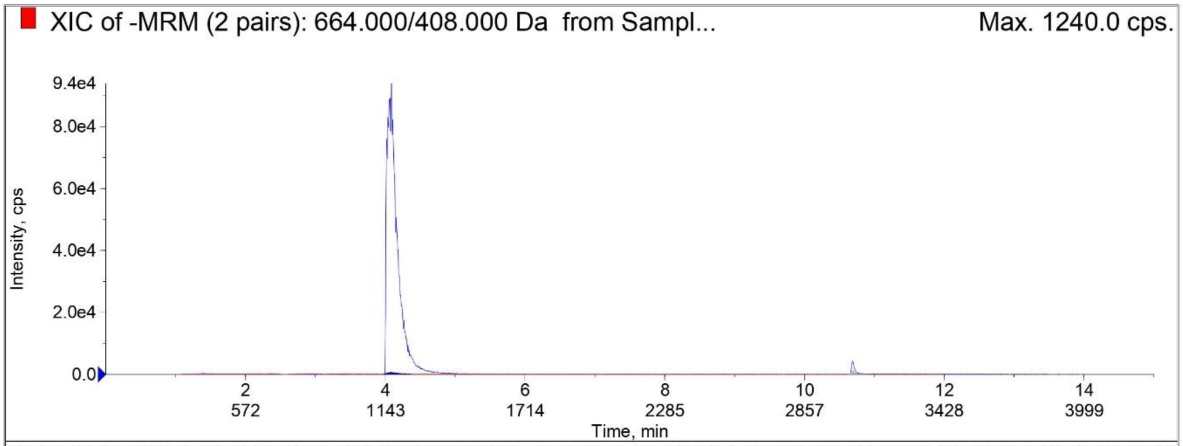

Peak List for "XIC of -MRM (2 pairs): 664.000/408.000 Da from Sample 17 (10:1 BORIC ACID 15 MIN -1) of NAD+CORMA1-0210

	Time (min)	Area (counts)	%Area	Height (cps)	%Height	Width (min)	Baseline Type
1	4.0758	6187.8700	56.2404	662.8929	35.4266	0.4027	Base to Base
2	10.6982	4814.6650	43.7596	1208.2818	64.5734	0.2591	Base to Base

***Figure S13b**. XIC of 10:1 Boric Acid:NAD^{+} at 15min detecting for NADH*

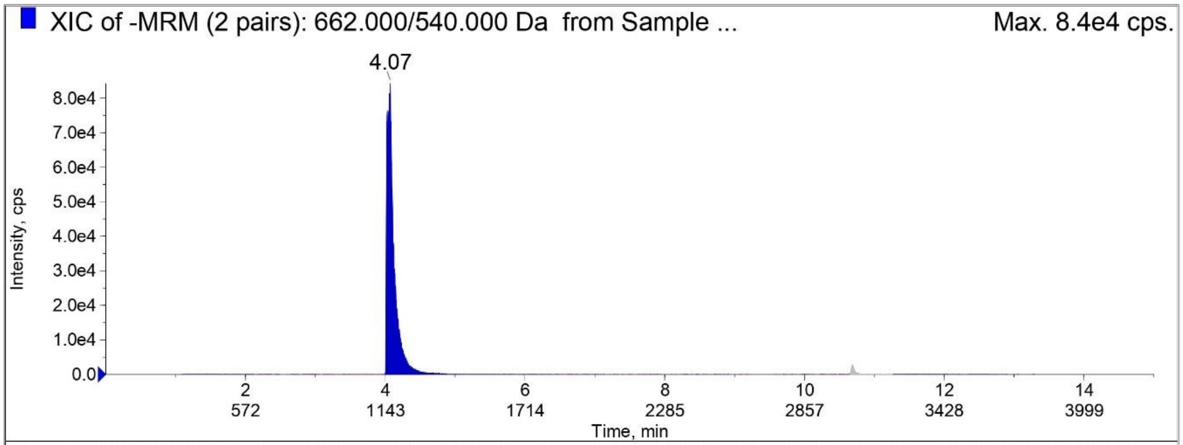

Peak List for "XIC of -MRM (2 pairs): 662.000/540.000 Da from Sample 18 (10:1 BORIC ACID 15 MIN -2) of NAD+CORMA1-0210:

	Time (min)	Area (counts)	%Area	Height (cps)	%Height	Width (min)	Baseline Type
1	4.0724	6.0858e5	98.2871	8.4305e4	96.7392	0.8860	Base to Base
2	10.6866	1.0606e4	1.7129	2841.7346	3.2608	0.6128	Base to Base

Figure S14a. *XIC of 10:1 Boric Acid:NAD$^+$ (2nd) at 15min detecting for NAD$^+$*

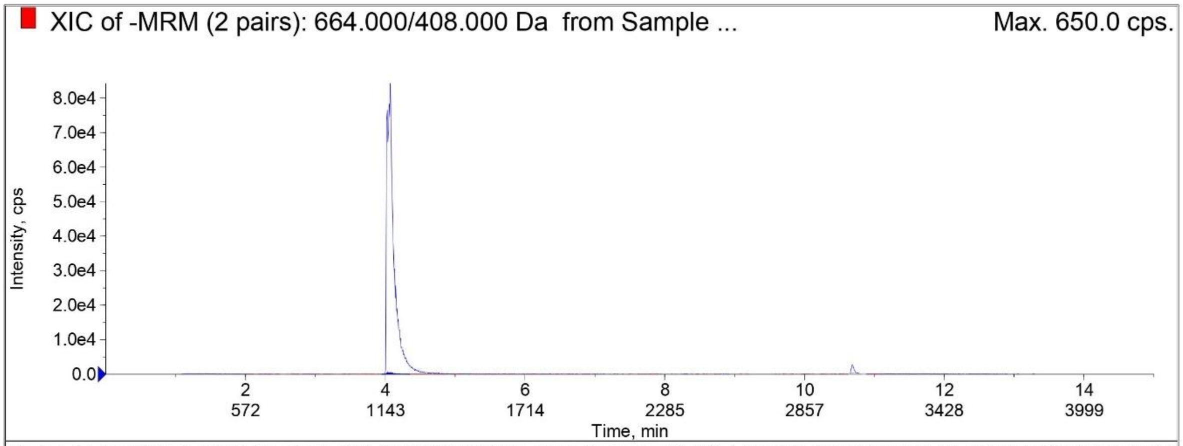

Peak List for "XIC of -MRM (2 pairs): 664.000/408.000 Da from Sample 18 (10:1 BORIC ACID 15 MIN -2) of NAD+CORMA1-0210:

	Time (min)	Area (counts)	%Area	Height (cps)	%Height	Width (min)	Baseline Type
1	4.0384	3332.3500	63.6310	663.5472	58.9562	0.3957	Base to Base
2	10.6773	1904.6400	36.3690	461.9441	41.0438	0.2521	Base to Base

Figure S14b. *XIC of 10:1 Boric Acid:NAD$^+$ (2nd) at 15min detecting for NADH*

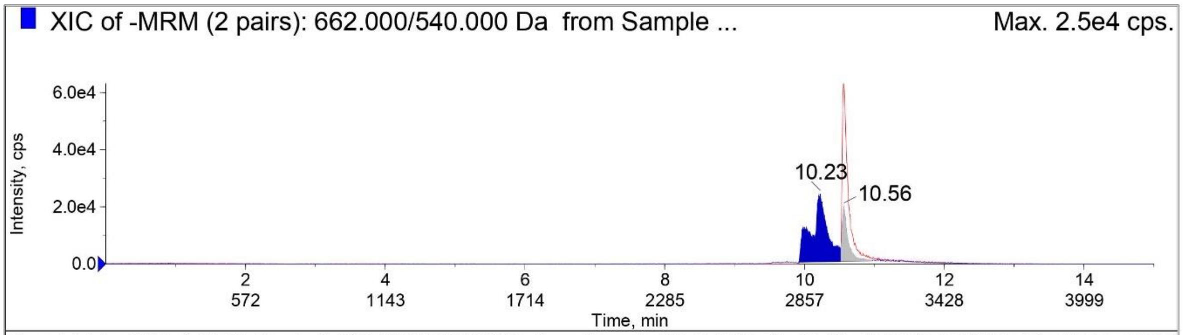

Peak List for "XIC of -MRM (2 pairs): 662.000/540.000 Da from Sample 25 (2-NAD+1mM-10mM-BH3-15MIN-THF) of NAD+CORM

	Time (min)	Area (counts)	%Area	Height (cps)	%Height	Width (min)	Baseline Type
1	10.2297	3.7304e5	77.3879	2.4046e4	55.0329	0.6023	Valley
2	10.5637	1.0900e5	22.6121	1.9648e4	44.9671	0.4447	Valley

Figure S15a. *XIC of 10:1 BH_3-THF:NAD^+ at 15min detecting for NAD^+*

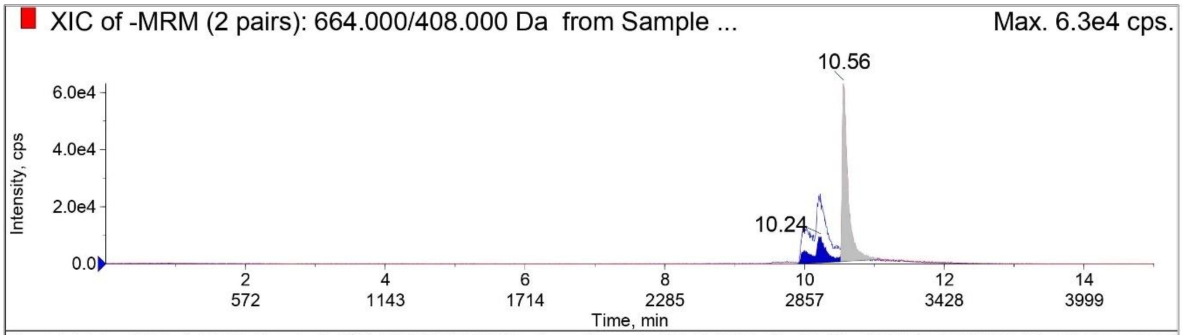

Peak List for "XIC of -MRM (2 pairs): 664.000/408.000 Da from Sample 25 (2-NAD+1mM-10mM-BH3-15MIN-THF) of NAD+CORM

	Time (min)	Area (counts)	%Area	Height (cps)	%Height	Width (min)	Baseline Type
1	10.2356	1.1967e5	24.8072	9047.6485	12.5333	0.6058	Valley
2	10.5597	3.6273e5	75.1928	6.3141e4	87.4667	0.5288	Valley

Figure S15b. *XIC of 10:1 BH_3-THF:NAD^+ at 15min detecting for NADH*

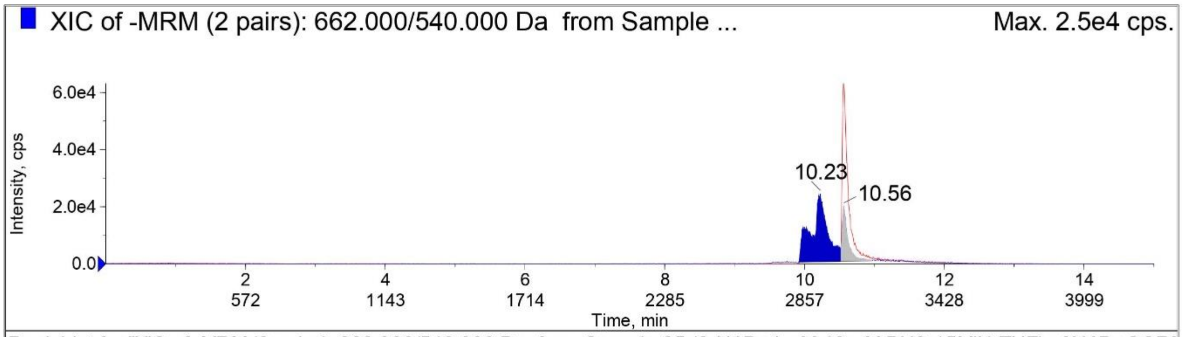

Peak List for "XIC of -MRM (2 pairs): 662.000/540.000 Da from Sample 25 (2-NAD+1mM-10mM-BH3-15MIN-THF) of NAD+CORM

	Time (min)	Area (counts)	%Area	Height (cps)	%Height	Width (min)	Baseline Type
1	10.2297	3.7304e5	77.3879	2.4046e4	55.0329	0.6023	Valley
2	10.5637	1.0900e5	22.6121	1.9648e4	44.9671	0.4447	Valley

***Figure S16a**. XIC of 10:1 BH_3-THF:NAD^+ (2nd) at 15min detecting for NAD^+*

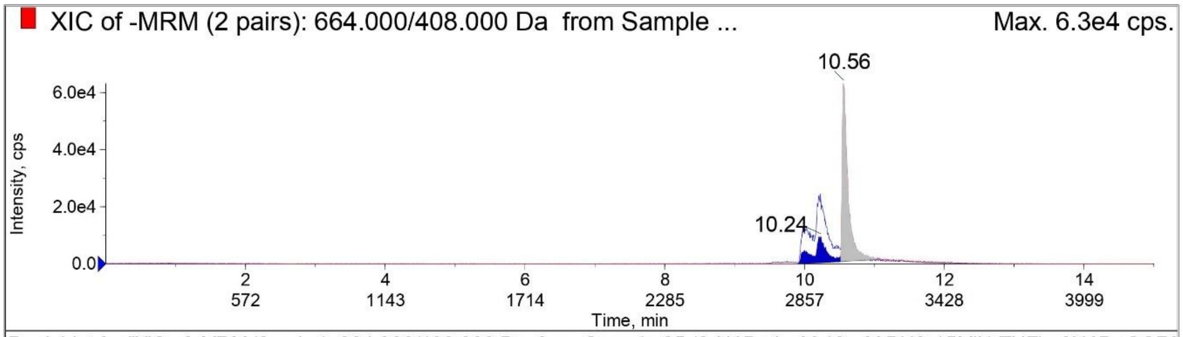

Peak List for "XIC of -MRM (2 pairs): 664.000/408.000 Da from Sample 25 (2-NAD+1mM-10mM-BH3-15MIN-THF) of NAD+CORM

	Time (min)	Area (counts)	%Area	Height (cps)	%Height	Width (min)	Baseline Type
1	10.2356	1.1967e5	24.8072	9047.6485	12.5333	0.6058	Valley
2	10.5597	3.6273e5	75.1928	6.3141e4	87.4667	0.5288	Valley

***Figure S16b**. XIC of 10:1 BH_3-THF:NAD^+ (2nd) at 15min detecting for NAD*H

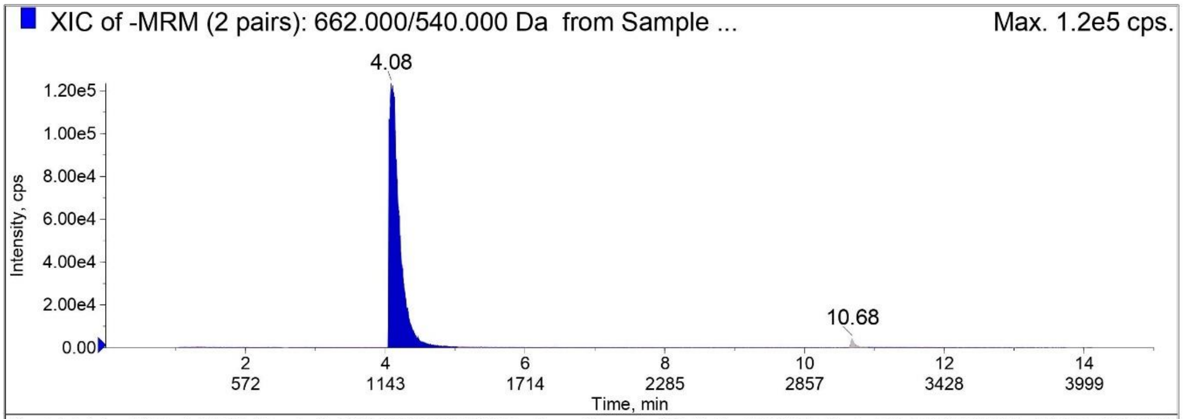

Peak List for "XIC of -MRM (2 pairs): 662.000/540.000 Da from Sample 29 (2-1mM NAD+ 2mL CO GAS 15MIN) of NAD+CORMA1

	Time (min)	Area (counts)	%Area	Height (cps)	%Height	Width (min)	Baseline Type
1	4.0840	1.2546e6	98.9143	1.2354e5	96.7142	1.0015	Base to Base
2	10.6846	1.3771e4	1.0857	4197.0230	3.2858	0.2592	Base to Base

Figure S17a. *XIC of 2 mL CO Gas in 1mM NAD^+ at 15min detecting for NAD^+*

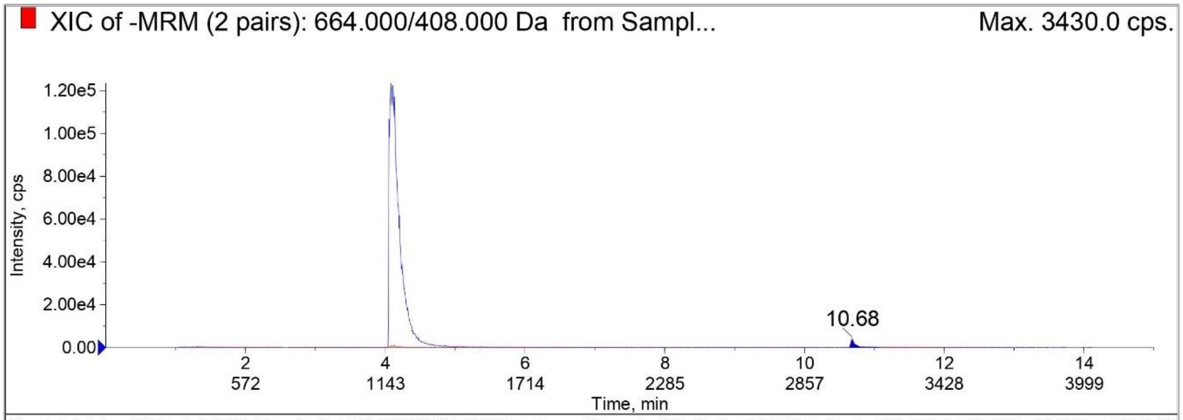

Peak List for "XIC of -MRM (2 pairs): 664.000/408.000 Da from Sample 29 (2-1mM NAD+ 2mL CO GAS 15MIN) of NAD+CORMA1

	Time (min)	Area (counts)	%Area	Height (cps)	%Height	Width (min)	Baseline Type
1	10.6833	1.5464e4	100.0000	3440.6667	100.0000	0.4307	Base to Base

Figure S17b. *XIC of 2 mL CO Gas in 1mM NAD^+ at 15min detecting for NADH*

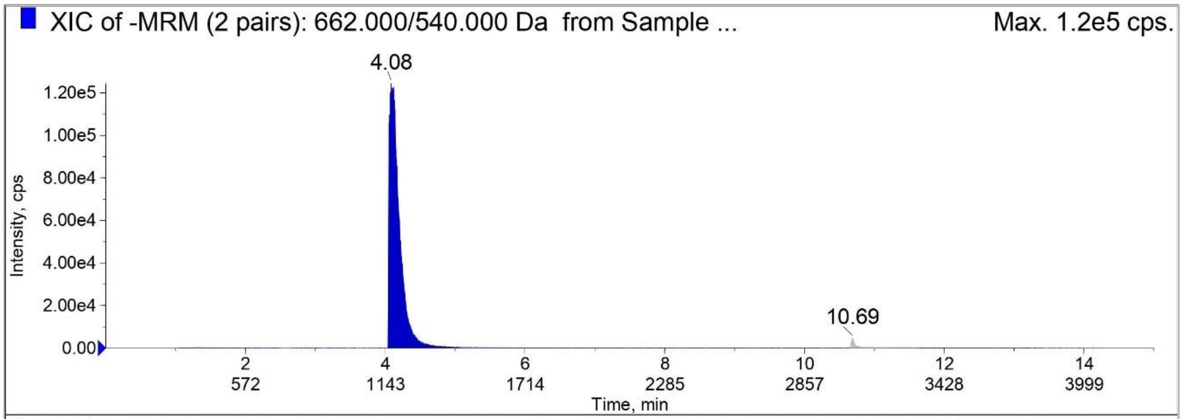

Peak List for "XIC of -MRM (2 pairs): 662.000/540.000 Da from Sample 30 (1-1mM NAD+ 2mL CO GAS 45MIN) of NAD+CORMA1

	Time (min)	Area (counts)	%Area	Height (cps)	%Height	Width (min)	Baseline Type
1	4.0838	1.2729e6	98.5302	1.2464e5	96.4877	1.0365	Base to Base
2	10.6853	1.8988e4	1.4698	4537.2798	3.5123	1.0015	Base to Base

Figure S18a. *XIC of 2 mL CO Gas in 1mM* NAD^+ *(2*nd*) at 45 min detecting for* NAD^+

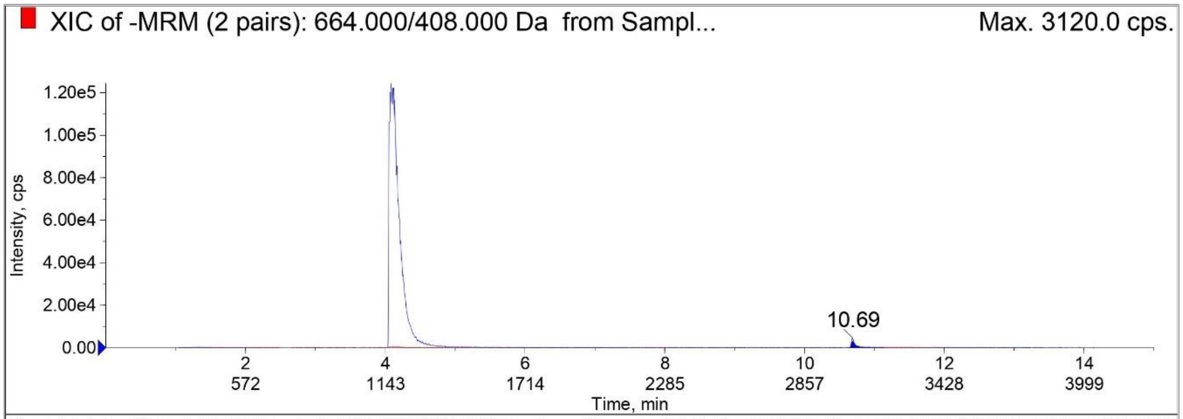

Peak List for "XIC of -MRM (2 pairs): 664.000/408.000 Da from Sample 30 (1-1mM NAD+ 2mL CO GAS 45MIN) of NAD+CORMA1

	Time (min)	Area (counts)	%Area	Height (cps)	%Height	Width (min)	Baseline Type
1	10.6909	1.3676e4	100.0000	3114.7059	100.0000	0.4903	Base to Base

Figure S18b. *XIC of 2 mL CO Gas in 1mM* NAD^+ *(2*nd*) at 45min detecting for NADH*

^{11}B NMR Spectra

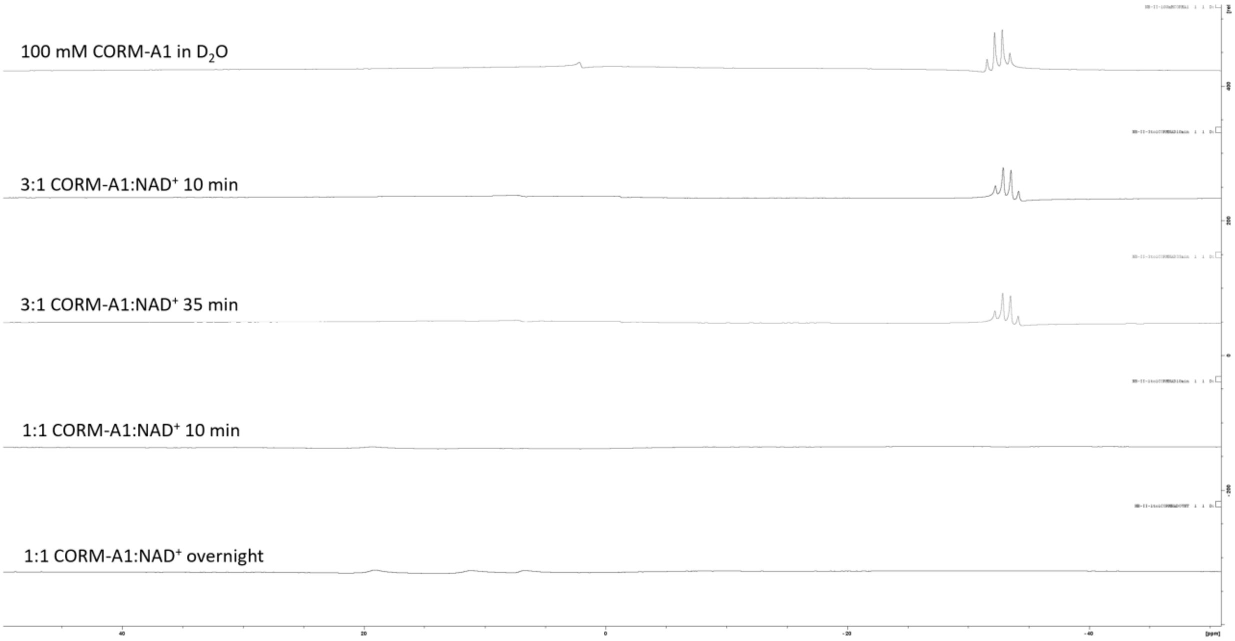

Figure S19. *^{11}B NMR of (from top to bottom): 100 mM CORM-A1 in D_2O , the reaction of 100 mM CORM-A1 and 33.3 mM NAD^+ at 10 minutes and 35 minutes , the preceeding reaction with an additional 66.6 mM NAD^+ added at 10 minutes and overnight*

SI References

1. Hofmann, T.; Held, S.; Lang, R.; Somoza, V. Pyrimidinium derivatives for use in the treatment or prevention of diabetes. WO 2010/055170 A1, 2010.
2. Sreeperumbuduru, R. S.; Abid, Z. M.; Claunch, K. M.; Chen, H.-H.; Mcgillivray, S. M.; Simanek, E. E., Synthesis and antimicrobial activity of triazine dendrimers with DABCO groups. *RSC Adv.* **2016,** *6* (11), 8806-8810.
